# The effects of respiratory rate and tidal volume on pulse pressure variation in healthy lungs–a generalized additive model approach may help overcome limitations

**DOI:** 10.1007/s10877-023-01090-6

**Published:** 2023-11-16

**Authors:** Johannes Enevoldsen, Birgitte Brandsborg, Peter Juhl-Olsen, Stephen Edward Rees, Henriette Vind Thaysen, Thomas W. L. Scheeren, Simon Tilma Vistisen

**Affiliations:** 1 Department of Clinical Medicine, Aarhus University, Aarhus, Denmark; 2 Department of Anaesthesiology, Aarhus University Hospital, Palle Juul-Jensens Boulevard 99, 8200 Aarhus, Denmark; 3 Department of Cardiothoracic- and Vascular Surgery, Anaesthesia Section, Aarhus University Hospital, Aarhus, Denmark; 4 Department of Health Science and Technology, Aalborg University, Aalborg, Denmark; 5 Department of Surgery, Aarhus University Hospital, Aarhus, Denmark; 6 Department of Anesthesiology, University of Groningen, University Medical Center Groningen, Groningen, The Netherlands; 7 Edwards Lifesciences, Irvine, USA

**Keywords:** Dynamic filling variable, Fluid responsiveness, Hemodynamic monitoring, Heart–lung interaction, Mechanical ventilation, Pulse pressure variation

## Abstract

Pulse pressure variation (PPV) is a well-established method for predicting fluid responsiveness in mechanically ventilated patients. The predictive accuracy is, however, disputed for ventilation with low tidal volume (V_T_) or low heart-rate-to-respiratory-rate ratio (HR/RR). We investigated the effects of V_T_ and RR on PPV and on PPV’s ability to predict fluid responsiveness. We included patients scheduled for open abdominal surgery. Prior to a 250 ml fluid bolus, we ventilated patients with combinations of V_T_ from 4 to 10 ml kg^−1^ and RR from 10 to 31 min^−1^. For each of 10 RR-V_T_ combinations, PPV was derived using both a classic approach and a generalized additive model (GAM) approach. The stroke volume (SV) response to fluid was evaluated using uncalibrated pulse contour analysis. An SV increase > 10% defined fluid responsiveness. Fifty of 52 included patients received a fluid bolus. Ten were fluid responders. For all ventilator settings, fluid responsiveness prediction with PPV was inconclusive with point estimates for the area under the receiver operating characteristics curve between 0.62 and 0.82. Both PPV measures were nearly proportional to V_T_. Higher RR was associated with lower PPV. Classically derived PPV was affected more by RR than GAM-derived PPV. Correcting PPV for V_T_ could improve PPV’s predictive utility. Low HR/RR has limited effect on GAM-derived PPV, indicating that the low HR/RR limitation is related to how PPV is calculated. We did not demonstrate any benefit of GAM-derived PPV in predicting fluid responsiveness.

Trial registration: ClinicalTrials.gov, reg. March 6, 2020, NCT04298931.

**Supplementary Information:**

The online version contains supplementary material available at 10.1007/s10877-023-01090-6.

## Background

Ventilator-induced pulse pressure variation (PPV) is a well-established and accurate method for predicting fluid responsiveness [1, 2]. Despite this accuracy, there are important limitations to its clinical use, including ventilation with low tidal volume (V_T_) and low heart-rate-to-respiratory-rate ratio (HR/RR) [3–5]. These limitations are frequently discussed, although their physiological basis is incompletely understood [3].

Low V_T_ ventilation was presented as a limitation by De Backer et al. in 2005, where it was shown that PPV only reliably predicted fluid responsiveness in patients ventilated with a V_T_ > 8 ml kg^−1^ [5]. However, low V_T_ was highly associated with a diagnosis of acute respiratory distress syndrome (ARDS), making it difficult to isolate the effect of V_T_. The study results may also have been affected by RR [4], respiratory system compliance [6], and other aspects of underlying lung disease. There are clinical studies with low V_T_ ventilation where PPV predicts fluid responsiveness well, but the predictive performance varies substantially [7]. It has been shown that PPV is highly correlated between different V_T_ settings in the same patient [8], and that low V_T_ is associated with lower PPV [9, 10]. Therefore, adjusting PPV by V_T_, respiratory driving pressure or changes in pleural pressure, has been suggested [11–13]. The effect of V_T_ on PPV is, however, still unclear. No studies have simultaneously investigated low V_T_ and low HR/RR limitations in the same patients to decouple these potentially interacting effects.

Low HR/RR was presented as a limitation by De Backer et al. in 2009, where it was shown in 17 patients that a HR/RR < 3.6 hindered accurate fluid responsiveness prediction using PPV [4]. The authors suggested that this was caused by a negative interference between the cyclic swings in the right and left ventricular stroke volume, respectively. We speculate that the low HR/RR limitation may, at least partially, result from a sampling problem specific to the classic PPV algorithm. In the HR/RR study [4], PPV was calculated for individual respiratory cycles [4, 14]. When there are few beats per respiratory cycle (low HR/RR), the beats may not lie close to the maximal and minimal possible pulse pressure (PP) in each cycle, giving an underestimation of PPV [10]. This limitation could be overcome by estimating PPV from a generalized additive model (GAM) of PP [15, 16].

We investigated the following research questions:

1. Predefined primary endpoint: how does altering V_T_ and RR affect PPV’s ability to predict fluid responsiveness?

2. What is the agreement between PPV derived with a GAM and with the classic approach, and is the agreement related to HR/RR?

3. How does altering V_T_ and RR affect PPV?

## Methods

This prospective study was conducted at Aarhus University Hospital, Denmark, after approval by the Central Denmark Region Ethics Committee (January 2020, case no.: 1-10-72-245-19) and registration on ClinicalTrials.gov (March 2020, identifier: NCT04298931). Patients gave written informed consent prior to participation.

### Study population

We included adults (≥ 18 years) scheduled for elective open abdominal surgery with hemodynamic monitoring using a FloTrac™ (4th generation) based device (EV1000™ or HemoSphere™, Edwards Lifesciences, Irvine, California).

Exclusion criteria were: irregular heart rhythm (e.g. atrial fibrillation), known left ventricular ejection fraction (LVEF) ≤ 40%, known right ventricular dysfunction (reported qualitatively or Tricuspid Annular Plane Systolic Excursion (TAPSE) < 17 mm) (a recent echocardiographic examination was *not* required), and pregnancy.

The cohort constitutes a convenience sample.

### Protocol

Anaesthesia was induced with propofol and maintained with sevoflurane on a Dräger Perseus® A500 (Dräger, Lübeck, Germany) anaesthesia machine; remifentanil was used for analgesia, and rocuronium for muscle relaxation. A thoracic epidural catheter was placed, and tested with 3 ml lidocaine 2% with adrenaline before induction. Arterial- and central venous pressure transducers were zeroed to atmospheric pressure and levelled at the right atrium. Patients were ventilated with pressure regulated volume control (VC-CMV + Autoflow®) with inspiration-expiration-ratio of 1:2, without any spontaneous breathing efforts.

**Fig. 1:**
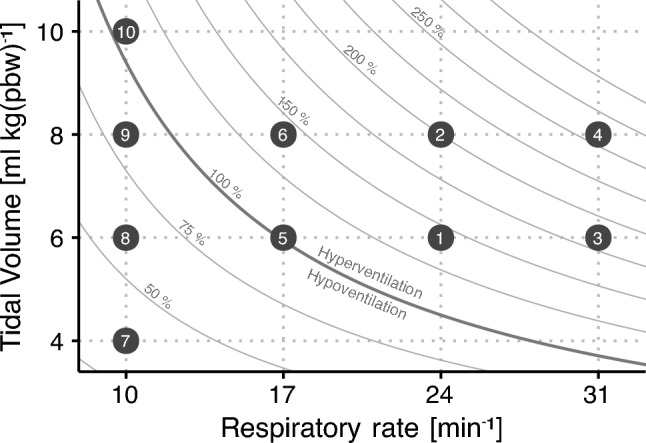
Example of the series of ventilator settings applied. Each numbered dot represents a combination of tidal volume (normalised to predicted body weight (pbw)) and respiratory rate. The numbers represent an example of the order of application of the settings. For each respiratory rate, tidal volumes were applied from low to high. Respiratory rates 17, 24 and 31 min^−1^ were applied in random order. Respiratory rate 10 min^−1^ was always applied last to avoid having any recruitment effect from the highest tidal volume (10 ml kg(pbw)^−1^) influence the remainder of the recording. Curved lines represent settings with equal alveolar ventilation, assuming a dead space volume of 1 ml kg(pbw)^−1^. The curved lines’ labels denote the alveolar ventilation relative to ventilation with a respiratory rate of 14 and a tidal volume of 7 ml kg(pbw)^−1^

Patients were observed in the context of a fluid administration prescribed by the treating anaesthetist, where acute hemodynamic perturbations were not expected (e.g. due to surgery). In the study period, any infusions were kept constant, and no bolus medication was administered. Before the fluid administration, a ventilation protocol was initiated comprised by a series of V_T_- and RR combinations (10 combinations of V_T_: 4, 6, 8, and 10 ml kg^−1^ (predicted body weight [17]) and RR: 10, 17, 24, and 31 min^−1^; see Fig. 1. Each setting was used for 30 s. For each RR, V_T_ was applied from lowest to highest. The order of the RR settings 17, 24 and 31 min^−1^ was randomised, while the four settings with RR of 10 min^−1^ were always applied last to minimise effects of potential lung recruitment from ventilation with V_T_ = 10 ml kg^−1^. The maximal allowed airway pressure was 40 cmH_2_O. Afterwards, the ventilator was reset to pre-protocol settings. Two to four minutes after the ventilation protocol, 250 ml of fluid (albumin or acetated Ringer’s solution as decided by the anesthesiologist) was administered through a fluid warming system (3 M™ Ranger™) over two minutes. The observation window ended two minutes after completion of the fluid administration.

### Data recording

We used VitalRecorder [18] to record data from the bedside monitor (Philips IntelliVue™ MX550, Eindhoven, the Netherlands) and the haemodynamic monitor (Hemosphere or EV1000), and VSCapture [19] to record data from the ventilator. Recordings were synchronised before analysis.

### Pulse pressure variation

Recordings were divided into ten 30-s windows: one for each protocolised ventilator setting (We used 30-s windows to have room to exclude e.g. ectopic beats. On pilot data, 20-s recordings were sufficient to derive PPV from a GAM). Individual heart beats were detected from the arterial waveform, and starttime (diastole) and PP (systolic pressure—diastolic pressure) were recorded. A beat was marked as an extrasystole and excluded if the time since the previous beat was less than 90% of the median of the ten preceding beat intervals. The following (post-ectopic) beat was also excluded. Additionally, outlier beats defined by a PP more than ± 25% from the median of the ten nearest beats were excluded. Thirty-second windows containing more than two extrasystoles were excluded.

**Fig. 2:**
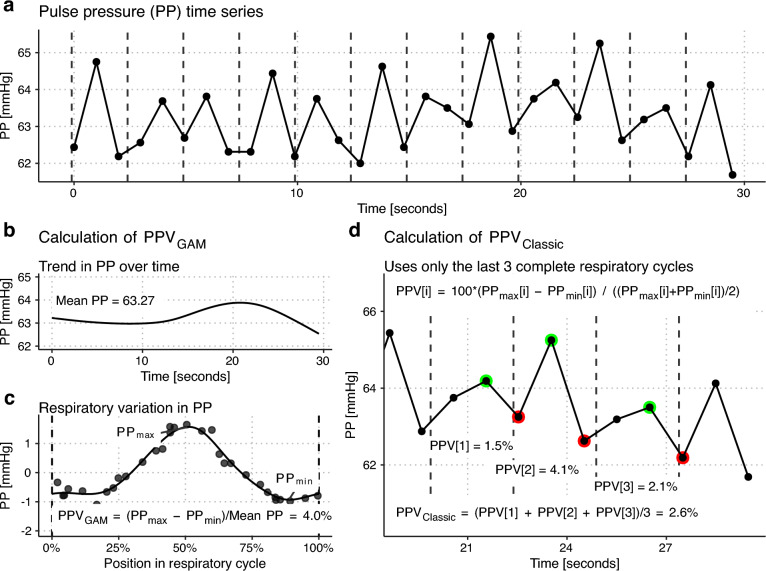
Illustration of the two methods used to calculate pulse pressure variation (PPV). The patient was ventilated with a tidal volume of 8 ml kg^−1^ and a respiratory rate of 24 min^−1^. Panel **a**: a 30 s time series of pulse pressure (PP) measurements is available for PPV calculation at each ventilator setting. Panels **b** and **c** illustrate calculation of PPV with a generalized additive model (GAM): the 30 s time series is decomposed into a trend in PP over time (**b**) and the cyclic variation in PP with each respiratory cycle (**c**). PPV_GAM_ is the variation in PP that is explained by the respiratory component (**c**). Panel **d** illustrates the classic calculation of PPV

Two PPV calculations were performed for each 30-s window: the classic calculation (PPV_Classic_) and PPV estimated using a GAM (see Fig. 2). With PPV_Classic_, we aimed to match the calculation of PPV described by De Backer et al. [4, 5]: Consecutive pairs of maximum and minimum PP were selected so each maximum is within one respiratory length of the previous minimum, and each minimum is within one respiratory length of the previous maximum. For each maximum-minimum pair, PPV was calculated as.

$$PPV\, = \,100 \cdot \frac{{~\left( {PP_{{max}} \, - \,PP_{{min}} } \right)}}{{~\left( {PP_{{max}} \, + \,PP_{{min}} } \right)/2}}$$

PPV_Classic_ was defined as the mean PPV of the last three maximum-minimum pairs during a protocol ventilator setting (see Fig. 2c).

The GAM-derived PPV (PPV_GAM_) was calculated as described previously (see Fig. 2b) [15]. This method quantifies the respiratory variation in PP by decomposing the series of PP measurements into a repeating respiratory component, and a slower trend over time. The respiratory component’s peak-to-peak amplitude divided by the mean PP constitutes PPV_GAM_.

### Fluid responsiveness

Stroke volume (SV) was estimated using pulse contour analysis (FloTrac). Each patient’s SV response to the 250 ml fluid challenge was calculated as:

$$\Delta SV\, = \,100 \cdot \frac{{SV_{{post}} \, - \,SV_{{pre}} }}{{SV_{{pre}} }}$$

Where SV_pre_ and SV_post_ are the medians of two minutes of SV measurements (six samples), before and after fluid administration. A $\Delta SV$>10% was considered a positive fluid response (prespecified).

### Statistics

Data were analysed with R 4.1.0, *tidyverse, pROC, boot* and *brms* [20–24]. Data and code for this section are available at https://doi.org/10.5281/zenodo.6984310.

#### Sample size calculation

The study was powered with respect to fluid responsiveness prediction. We expected that 50% of patients would be fluid responders; therefore, to reach a power of 0.9 with α = 0.05, 33 patients should be included. We decided to include 50 patients to account for uncertainty in the number of fluid responders and to increase precision of the mixed-effects model estimates.

#### Fluid responsiveness prediction

Fluid responsiveness prediction, with PPV_GAM_ or PPV_Classic_ as predictors, was evaluated using receiver operating characteristic (ROC) analysis. Confidence intervals (95%) for area under the ROC curve (AUROC) were calculated using the DeLong-method [25].

#### Comparison of PPV_Classic_ and PPV_GAM_

We used Bland–Altman analysis to compare PPV_Classic_ with PPV_GAM_ at each ventilator setting [26]. Limits of agreement (95% LoA) were calculated as mean(PPV_GAM_ − PPV_Classic_) ± 1.96*SD(PPV_GAM_ − PPV_Classic_), where SD is the standard deviation. Confidence intervals (95%) for bias and LoA were calculated using nonparametric bootstrapping with 4000 resamples.

#### The effect of V_T_ and RR on PPV

To investigate the effect of each ventilator setting on PPV, we fitted a Bayesian mixed-effects model with a patient-specific intercept and a separate variance for each ventilator setting.

In brief, the model describes the relative effects of V_T_ and RR on PPVs measured within a patient. The relative effects were fitted for each of the two PPV methods (GAM and Classic). While the estimated relative differences were the same for all patients (fixed effects), the absolute PPV values can differ between patients (random effect). The physiological understanding of this is that patients have individual Frank-Starling curve operating positions, and that each ventilator setting produces a PPV conditional on the patient’s position on the Frank-Starling curve.

To increase robustness to outlying values, a Student’s T likelihood distribution with four degrees of freedom was used. The link function was the logarithm, and, consequently, the exponential of the model coefficients are the relative effects on PPV.

A formal model formulation with prior specification and rationale is presented in Online Resource 1.

The model was sampled using Stan, via the R interface *brms* [24, 27].

Posterior distributions were summarised as median and 95% interval (2.5th to 97.5th percentile). This interval gives a range of values for each parameter that are compatible with the observed data, similar to a confidence interval [28].

To compare residual standard deviation across different ventilator settings, we calculated the coefficient of variation (CV): the residual standard deviation divided by the expected value of PPV for each ventilator setting.

## Results

From May 2020 to June 2021, we included 52 patients who underwent open abdominal surgery under general anaesthesia. Of these, 50 had a successful measurement of the response to the 250 ml fluid challenge and were eligible for fluid responsiveness analysis. The mean duration of the fluid infusion was 113 (SD 27) seconds. Ten patients were fluid responders (ΔSV > 10%). Patient characteristics, vasopressor use and average SV response to fluid are shown in Table 1.

**Table 1:** Characteristics of patients included in the study

Variable	ΔSV ≤ 10%, N = 40^1^	ΔSV > 10%, N = 10^1^	Total, N = 52^2^
Age	65 [57, 73]	61 [55, 69]	64 [57, 72]
Sex			
Female	21 (52%)	4 (40%)	26 (50%)
Male	19 (48%)	6 (60%)	26 (50%)
Height [cm]	173 [166, 179]	180 [165, 184]	173 [165, 180]
Weight [kg]	78 [70, 82]	78 [62, 87]	79 [70, 84]
Body mass index	24.8 [23.4, 28.2]	22.8 [21.6, 25.0]	24.7 [23.3, 28.2]
Predicted body weight (pbw, kg)	67 [58, 74]	75 [57, 79]	68 [57, 75]
Known hypertension	17 (42%)	1 (10%)	19 (37%)
ASA score			
1	14 (35%)	6 (60%)	21 (40%)
2	22 (55%)	3 (30%)	25 (48%)
3	3 (7.5%)	1 (10%)	5 (9.6%)
4	1 (2.5%)	0 (0%)	1 (1.9%)
Surgical procedure^3^			
HIPEC	27 (68%)	7 (70%)	36 (69%)
APE and/or VRAM	7 (18%)	1 (10%)	8 (15%)
Colon resection	2 (5.0%)	0 (0%)	2 (3.8%)
Other	4 (10%)	2 (20%)	6 (12%)
Fluid type for fluid challenge			
Acetated Ringer's solution	30 (75%)	6 (60%)	36 (69%)
Human albumin	10 (25%)	4 (40%)	14 (27%)
No fluid challenge	0 (0%)	0 (0%)	2 (3.8%)
Any vasopressor during study protocol	36 (90%)	10 (100%)	47 (90%)
Noradrenaline rate [µg kg^−1^ min^−1^]			
0	14 (35%)	6 (60%)	21 (40%)
< 0.1	19 (48%)	4 (40%)	23 (44%)
≥ 0.1	7 (18%)	0 (0%)	8 (15%)
Dopamine rate [µg kg^−1^ min^−1^]			
0	22 (55%)	4 (40%)	28 (54%)
< 5	17 (42%)	5 (50%)	22 (42%)
≥ 5	1 (2.5%)	1 (10%)	2 (3.8%)
Pre-intervention ventilation			
Tidal volume [ml kg^−1^ (pbw)]	7.2 [6.9, 7.8]	6.8 [6.4, 8.0]	7.2 [6.9, 7.8]
Respiratory rate [min^−1^]	14 [14, 16]	14 [14, 16]	14 [14, 16]
Positive end expiratory pressure [cmH_2_0]	5 [5, 6]	6 [5, 6]	5 [5, 6]
Fluid challenge			
SV before fluid challenge [mL]	71 [60, 82]	58 [54, 66]	68 [59, 81]
SV response to fluid challenge [%]	2.1 [0.6, 5.4]	17.3 [13.7, 20.1]	3.8 [1.0, 8.9]
MAP before fluid challenge [mmHg]	70 [64, 74]	68 [62, 73]	69 [63, 74]
MAP after fluid challenge [mmHg]	78 [71, 82]	74 [68, 81]	77 [70, 83]
SAP before fluid challenge [mmHg]	106 [96, 120]	99 [90, 111]	105 [95, 119]
SAP after fluid challenge [mmHg]	118 [106, 130]	112 [108, 127]	118 [107, 129]
DAP before fluid challenge [mmHg]	53 [49, 57]	55 [50, 59]	53 [49, 58]
DAP after fluid challenge [mmHg]	57 [53, 63]	56 [51, 65]	57 [52, 64]
PP before fluid challenge [mmHg]	54 [48, 66]	46 [42, 60]	53 [44, 65]
PP after fluid challenge [mmHg]	60 [53, 73]	60 [50, 67]	60 [52, 71]

*ASA score* American Society of Anesthesiologists physical status classification system, *SV* Stroke volume estimate from FloTrac® pulse contour analysis, *MAP* Mean arterial pressure, *SAP* Systolic arterial pressure, *DAP* Diastolic arterial pressure, *PP* Arterial pulse pressure, *HIPEC* Hyperthermic intraperitoneal chemotherapy, *APE* Abdomino-perineal excision, *VRAM* Vertical rectus abdominis myocutaneous flap, *pbw* predicted body weight

^1^Median [IQR]; n (%), ^2^Two patients received no fluid challenge, ^3^Surgical procedures are counted in the first matching category

Four of the 52 patients eligible for PPV analysis reached (or were close to) the maximal allowed airway pressure (40 cmH_2_0) at RR = 31 min^−1^, V_T_ = 6 ml kg^−1^, and did not have the setting: RR = 31 min^−1^, V_T_ = 8 ml kg^−1^ applied. In nine windows (ventilator settings), there were more than two extrasystoles, leaving 507 of the 520 potential windows available for analysis. Online Resource 2 shows included and excluded beats and PPV calculation for all ten ventilator settings in all patients.

### Fluid responsiveness prediction

**Fig. 3:**
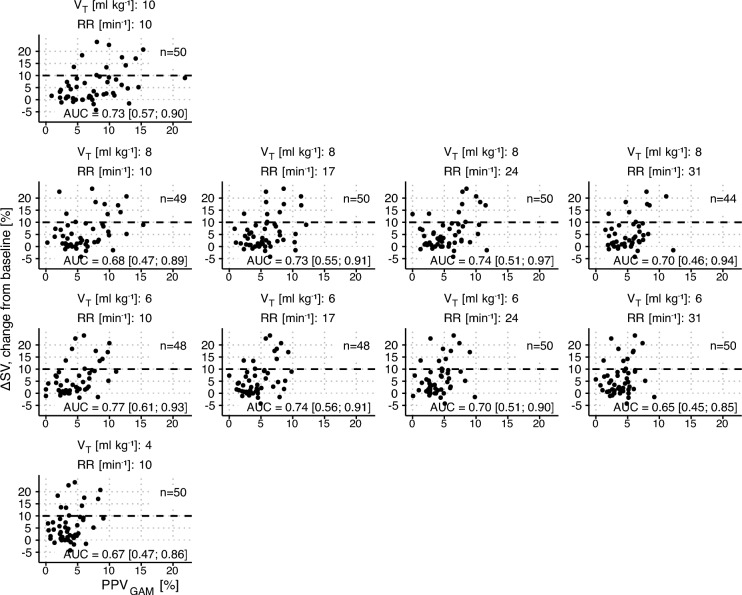
Scatter plots of the relation between PPV derived from a generalized additive model (PPV_GAM_) and the stroke volume response (ΔSV) to a 250 ml fluid challenge. Panels are arranged with tidal volumes (V_T_) in rows and respiratory rates (RR) in columns. One fluid challenge was evaluated for each patient (n = 50), while PPV_GAM_ was calculated for each of the 10 ventilator settings

Figure 3 shows scatter plots of PPV_GAM_ and the corresponding fluid response (ΔSV) for all ventilator settings (a similar figure for PPV_Classic_ is available in Online Resource 3 Fig. S1). The capacities of PPV_GAM_ and PPV_Classic_ to classify fluid responsiveness (ΔSV > 10%) are presented as ROC curves in Online Resource 3 Fig. S2. At the ventilator setting RR = 10 min^−1^, V_T_ = 10 ml kg^−1^, PPV_GAM_ had an area under the ROC curve (AUC) of 0.73 (95% CI 0.57 to 0.90), while the AUC for PPV_Classic_ was 0.74 (95% CI 0.57 to 0.92). At RR = 31 min^−1^, V_T_ = 6 ml kg^−1^, PPV_GAM_ had an AUC of 0.65 (95% CI 0.45 to 0.85), while the AUC for PPV_Classic_ was 0.62 (95% CI 0.40 to 0.84). Online Resource 3 Table S1 presents AUC, optimal PPV threshold, sensitivity and specificity for fluid responsiveness discrimination for all ten ventilator settings.

### Comparison of PPV_Classic_ and PPV_GAM_

**Fig. 4:**
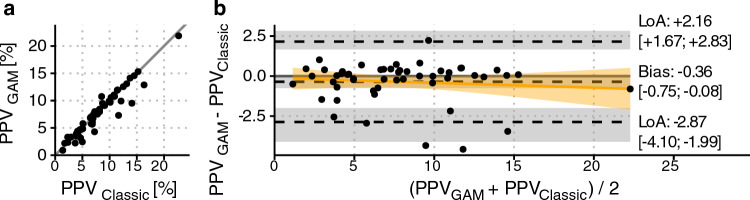
Scatter plot (**a**) and Bland–Altman plot (**b**) showing the relation between pulse pressure variation derived from a generalized additive model (PPV_GAM_) and pulse pressure variation calculated using the classic approach (PPV_Classic_) when tidal volume is 10 ml kg^−1^ and respiratory rate is 10 min^−1^. In panel b, the outer dashed lines represent 95% limits of agreement (LoA). Grey areas are 95% confidence intervals for bias and LoA. The yellow line and area is a linear regression fit with 95% confidence intervals

At ventilator setting RR = 10 min^−1^, V_T_ = 10 ml kg^−1^, PPV_GAM_ was, on average, slightly lower than PPV_Classic_: mean difference (bias) = −0.36 (95% CI −0.75 to −0.08); limits of agreement (95%) were −2.87 to 2.16 (see Fig. 4). Bland–Altman plots comparing PPV_Classic_ and PPV_GAM_ for all ten ventilator settings are presented in Online Resource 3 Fig. S3.

**Fig. 5:**
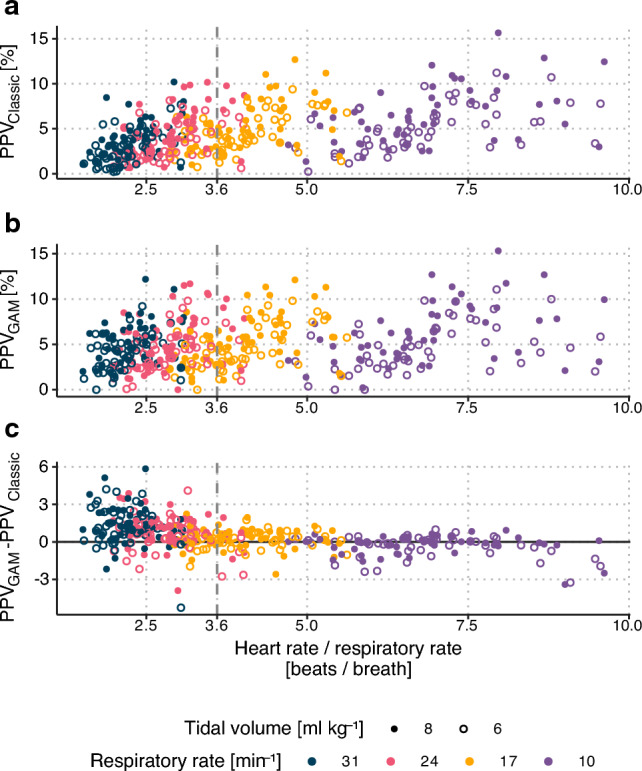
The relationship between heart rate/respiratory rate and PPV when PPV is calculated using the classic approach (**a**) and using a generalized additive model (GAM) (**b**). Panel **c** shows the difference between PPV_GAM_ and PPV_Classic_ calculated on the same data (403 total observations in 50 patients)

The relationship between PPV and HR/RR is shown in Fig. 5. At HR/RR below 3.6, PPV_GAM_ was generally higher than PPV_Classic_: bias = 0.93 (95% CI 0.76 to 1.11); limits of agreement (95%) were −1.73 to 3.59. At HR/RR above 3.6, PPV_GAM_ and PPV_Classic_ gave very similar values: bias = −0.09 (95% CI −0.23 to 0.03); limits of agreement (95%) was −1.85 to 1.67.

### The effect of *V*_T_ and RR on PPV

**Fig. 6:**
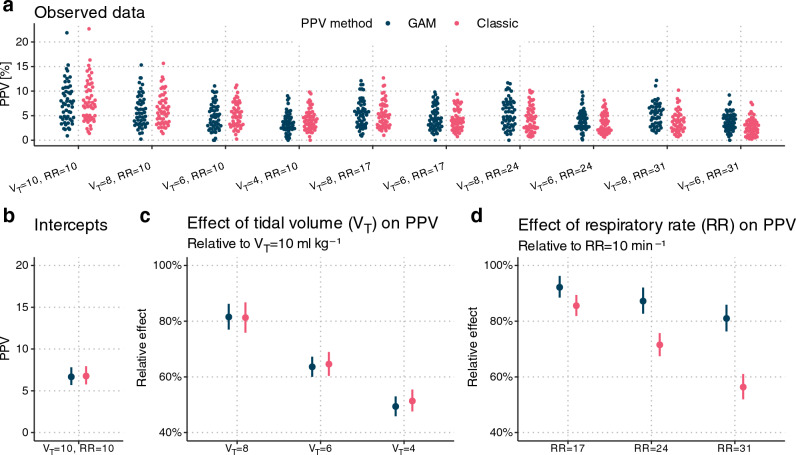
Parameter estimates for a Bayesian mixed-effects model, describing the effect of tidal volume (V_T_) and respiratory rate (RR) on pulse pressure variation (PPV). Parameters are estimated for both PPV derived using a generalized additive model (GAM) and using a classic approach (Classic). Panel **a** presents the observed PPV values (outcomes) using each method (n = 507 for both GAM and Classic). Panel **b**, **c** and **d** present parameter estimates. Vertical bars are 95% compatibility intervals

Model parameters are shown in Fig. 6. Estimates of the effects of V_T_ = 10, 8 or 6 ml kg^−1^ were very close to a direct proportionality between V_T_ and PPV for both PPV_Classic_ and PPV_GAM_. Relative to PPV_GAM_ at V_T_ = 10 ml kg^−1^, PPV_GAM_ at V_T_ = 8 ml kg^−1^ was 81 (95% CI 77 to 86)% and PPV_GAM_ at V_T_ = 6 ml kg^−1^ was 64 (95% CI 60 to 67)%. At V_T_ = 4 ml kg^−1^, PPV_GAM_ was 49 (95% CI 46 to 53)% and not compatible with the 40% expected from a direct proportionality between V_T_ and PPV. The effect of V_T_ on PPV_Classic_ was similar.

Higher RR was associated with lower PPV, and the effect was most pronounced for PPV_Classic_: at RR = 31 min^−1^, PPV_Classic_ was 56 (95% CI 52 to 61)% and PPV_GAM_ was 81 (95% CI 76 to 86)%, both relative to at RR = 10 min^−1^.

The residual variation is shown in Online Resource 3 Fig. S4. The relative variation of the observations around the model predictions (CV of the residuals) was similar between PPV_Classic_ and PPV_GAM_, except for at RR = 31 min^−1^, where PPV_Classic_ had a higher uncertainty (the difference in CV is 16 (95% CI 3 to 30)%-points). The CV was lowest at RR = 17 min^−1^ for both PPV_Classic_ and PPV_GAM_.

## Discussion

This study had three main aims. First, we sought to describe V_T_’s and RR’s impact on PPV’s ability to predict fluid responsiveness. Second, we compared PPV calculated with a classical approach to PPV calculated from a GAM, as we expected that the classical approach tends to underestimate PPV at low HR/RR. Third, we investigated the direct impact of V_T_ and RR on PPV.

### Fluid responsiveness prediction

Unfortunately, not much can be derived about PPV’s ability to predict fluid responsiveness from this study, mainly due to the low number of responders. Point estimates for both PPV_GAM_ and PPV_Classic_ showed fluid responsiveness prediction with mediocre/poor accuracy, even when patients were ventilated at V_T_ = 10 ml kg^−1^ and RR = 10 min^−1^. Most confidence intervals were compatible with AUCs from 0.6 to 0.9 (poor to excellent classification).

Based on basic Bland–Altman analysis (Online Resource 3 Fig. S3), we demonstrated that PPV derived from GAM and the classic approach are very similar at V_T_ ≥ 6 ml kg^−1^ and RR ≤ 17 min^−1^.

### Comparison of PPV_Classic_ and PPV_GAM_ and the effects of V_T_ and RR on PPV

Based on basic Bland–Altman analysis (Online Resource 3 Fig. S3), we demonstrated that PPV derived from GAM and the classic approach are very similar at V_T_ ≥ 6 ml kg^−1^ and RR ≤ 17 min^−1^.

The mixed-effects model demonstrated that within the same patient, PPV was nearly proportional to V_T_ across various levels of V_T_ and RR. The V_T_ effect was similar for PPV_GAM_ and PPV_Classic_ (see Fig. 6). Reuter et al. 2003, reported a similar proportionality between V_T_ and stroke volume variation, while V_T_’s effect on PPV was clear, but less than proportional [9]. Liu et al. 2016 report results that seem to be compatible with a proportionality between V_T_ and PPV, though they did not directly analyse this relationship [8]. High RR reduced PPV_Classic_ markedly more than it reduced PPV_GAM_ (see Fig. 6). This difference likely reflects a sampling effect rather than a physiological effect. At high RR, PPV_Classic_ is calculated from very few beats per respiratory cycle, increasing the risk of a falsely low PPV. This result is in accordance with the original study investigating the HR/RR ratio effect, which found that PPV (corresponding to PPV_Classic_ in the present paper) dropped markedly when the HR/RR was < 3.6 [4]. Conversely, PPV_GAM_ works well in low HR/RR situations, since it combines information from several respiratory cycles (see Fig. 2). This difference between PPV_Classic_ and PPV_GAM_ at low HR/RR is also illustrated in Fig. 5. When HR/RR is an exact low integer ratio (e.g., one, two or three beats per ventilation), both PPV_GAM_ and PPV_Classic_ can underestimate preload responsiveness, since the beats will occur at constant positions in each respiratory cycle, and these positions may not represent the minimum and maximum preload induced by the ventilation.

The model allows us to account for ventilator settings in the interpretation of PPV. As an example, consider a patient ventilated at a V_T_ of 6 ml kg^−1^ and RR of 24 min^−1^. We estimate a PPV of 8% with the GAM method. The best guess of what PPV would be if V_T_ is changed to 10 ml kg^−1^ and RR to 10 min^−1^ (a setting where an optimal PPV threshold seems established [2]), is then

$$PPV_{{RR = 10,V_T = 10}} \, = \,8\% \cdot \frac{1}{{0.64}} \cdot \frac{1}{{0.87}} \approx 14\%$$

For PPV_GAM_, a pragmatic bedside approximation would be to consider PPV directly proportional to V_T_ and disregard the effect of RR:

$$PPV_{{RR = 10,V_T = 10}} \, = \,PPV \cdot \frac{{10}}{{V_{T} }}$$

Where PPV is the current PPV and V_T_ is the current V_T_ in ml kg(pbw)^−1^. This approximation works because for reciprocal changes in RR and V_T_ (approximately maintaining minute ventilation), the overcorrection from considering the effect of V_T_ as proportional, closely matches the effect of the RR change.

The CV was similar for PPV_GAM_ and PPV_Classic_, except when RR was high. Here PPV_Classic_ had a significantly higher CV. This is in accordance with the sampling problem affecting PPV_Classic_ described above.

### Limitations

This study included relatively few fluid responders (20%). The aim of including patients with no acute need for intervention during the 6-to-8-min ventilation protocol may have resulted in a more fluid-optimised population. Also, the uncalibrated pulse contour analysis estimate of CO is clinically acceptable and is probably one of the most used CO modalities in GDT protocols [29], but it is not the gold standard for measuring CO.

Regardless of the cause, the poor predictive performance of PPV precludes meaningful investigation of the hypothesised advantage of using PPV_GAM_ to predict fluid responsiveness at low HR/RR. Also, PPV_GAM_ is currently not available on commercial clinical monitors, but could be implemented if future studies demonstrate a clinical advantage of the method.

We do not investigate the effect of heart rate (HR) on PPV. Any effect of low HR/RR on PPV could be caused, in part, by a direct effect of HR. In our data, higher HR is associated with higher PPV (see Fig. 5a and b), but we cannot say anything about causality, since we do not experimentally control HR. It may be that both HR and PPV are associated with e.g. volume status.

In accordance with fluid responses, PPV values were relatively low. We do not know whether the relative effects of RR and V_T_ found in this study also apply to patients with higher PPV.

Patients eligible for major abdominal surgery are generally in good cardiopulmonary condition. Additionally, we excluded subjects with LVEF ≤ 40%, right ventricular dysfunction or arrhythmia. The results may not generalise to a population with different HR, cardiac function or lung compliance, such as ICU patients with ARDS, or other critically ill patients.

## Conclusion

We demonstrate that the current understanding of ventilator settings’ impact on PPV is insufficient. The limitation associated with low HR/RR seems to be predominantly related to a specific method of deriving PPV rather than a physiological limitation. At high RRs PPV should be estimated over multiple respiratory cycles to avoid a basic sampling problem. Also, PPV is nearly proportional to V_T_, suggesting that correcting PPV for V_T_ might make the optimal threshold less dependent on V_T_, thus improving the utility of PPV. However, it was not possible to demonstrate whether PPV based on GAM modelling would result in a better prediction of fluid responsiveness than the classical method of deriving PPV.

### Supplementary Information

Below is the link to the electronic supplementary material.

Supplementary file1 (PDF 2911 KB)

Supplementary file2 (PDF 2678 KB)

Supplementary file3 (PDF 237 KB)
