# Supplementary material for: The effects of respiratory rate and tidal volume on pulse pressure variation in healthy lungs–a generalized additive model approach may help overcome limitations
Source: J Clin Monit Comput. 2023 Nov 16;38(1):57–67. doi: 10.1007/s10877-023-01090-6 (PMC10879304; doi:10.1007/s10877-023-01090-6)
Supplement: Supplementary file 1 — Supplementary file1 (PDF 2911 KB) [file 10877_2023_1090_MOESM1_ESM.pdf]

Bayesian Mixed-Effects model of Pulse Pressure Variation by Tidal Volume and Respiratory Rate

This document presents code for fitting, analysing and visualising the Bayesian mixed-effects model presented in the paper. It includes a presentation of model priors, with arguments for why they are considered weakly informative.

1 Setup

```
library(tidyverse)
library(patchwork) # For combining plots

library(brms)      # For fitting Stan models
library(tidybayes) # For working with fitted Stan models
options(mc.cores = parallel::detectCores())

source("plot_settings.R") # Plot theme and utility functions
theme_set(theme_paper())
```

2 Load data

Data is shared in the `data/` folder in the code repository:

https://doi.org/10.5281/zenodo.6984310

A codebook is available in the same folder.

```
PPV_df <- read_csv("data/vent_setting_study-vent_protocol.csv") |>
  mutate(
    # Create factors for ventilator settings
    id_f = factor(id),
    vent_rel_vt_f = factor(vent_rel_vt, levels = c(10, 8, 6, 4)),
    vent_RR_f = factor(vent_RR, levels = c(10, 17, 24, 31)),
    vent_setting = interaction(vent_rel_vt, vent_RR, drop = TRUE) |>
      forcats::fct_relevel("10.10", "8.10", "6.10", "4.10",
                           "8.17", "6.17",
                           "8.24", "6.24",
                           "8.31", "6.31")
  ) |>
```

```
  # Remove the 13 / 520 rows without a PPV value. PPV is missing either because the
  # ventilator setting was not applied or because PPV estimation was infeasible
  # because of frequent extra-systoles (≥3 in one window).
  drop_na(PPV_gam)

# Labels for vent settings
vent_setting_levels <- c(
  "10.10" = "V<sub>T</sub>=10, RR=10",
  "8.10" = "V<sub>T</sub>=8, RR=10",
  "6.10" = "V<sub>T</sub>=6, RR=10",
  "4.10" = "V<sub>T</sub>=4, RR=10",
  "8.17" = "V<sub>T</sub>=8, RR=17",
  "6.17" = "V<sub>T</sub>=6, RR=17",
  "8.24" = "V<sub>T</sub>=8, RR=24",
  "6.24" = "V<sub>T</sub>=6, RR=24",
  "8.31" = "V<sub>T</sub>=8, RR=31",
  "6.31" = "V<sub>T</sub>=6, RR=31"
)

vt_levels <- c(
  "10" = "V<sub>T</sub>=10",
  "8" = "V<sub>T</sub>=8",
  "6" = "V<sub>T</sub>=6",
  "4" = "V<sub>T</sub>=4"
)

rr_levels <- c(
  "10" = "RR=10",
  "17" = "RR=17",
  "24" = "RR=24",
  "31" = "RR=31"
)

# Pivot PPV data frame to long format with one column for PPV
# and one column indicating the method (Classic or GAM)
PPV_df_long <- PPV_df |>
  pivot_longer(c(PPV_gam, PPV_classic),
               values_to = "PPV",
               names_to = "PPV_method",
               names_prefix = "PPV_") |>
  mutate(PPV_vt = 10*PPV/vent_rel_vt,
         label = vent_setting_levels[as.character(vent_setting)] |>
           factor(levels = vent_setting_levels),
         PPV_method = factor(PPV_method, levels = c("gam", "classic")))
```

3 Model specification

The model (m1), fitted with brms, corresponds to the following model in mathematical notation:

$$
\begin{aligned}
& \textbf{[Likelihood]} \\
PPV_{strm} &\sim StudentT(\mu_{strm}, \sigma_{trm}, \text{df} = 4) \\
& \textbf{[Linear model of } log(\mu)\textbf{]} \\
log(\mu_{strm}) &= \beta 0_m + \beta 1_{tm} + \beta 2_{rm} + \alpha_s \\
& \textbf{[Addaptive prior for random effect of subject]} \\
\alpha_s &\sim Normal(0, \sigma_\alpha) \\
& \quad \text{, for subject s = 1, ... ,52} \\
& \textbf{[Prior for SD of subjects]} \\
\sigma_\alpha &\sim truncNormal(0, 1.5, low = 0) \\
& \textbf{[Prior for PPVmethod-specific intercept]} \\
\beta 0_m &\sim Normal(2.3, 1) \\
& \quad \text{, for PPVmethod m = (gam, classic)} \\
& \textbf{[Prior for } \beta \textbf{]} \\
(\beta 1_{tm}, \beta 2_{rm}) &\sim Normal(0, 2) \\
& \quad \text{, for ventVT t = (8,6,4); ventRR r = (17,24,31); PPVmethod m = (gam, classic)} \\
& \textbf{[Linear model of } log(\sigma)\textbf{]} \\
log(\sigma_{trm}) &= \gamma 0_m + \gamma 1_{tm} + \gamma 2_{rm} \\
& \textbf{[Prior for } \gamma \textbf{]} \\
(\gamma 0_m, \gamma 1_{tm}, \gamma 2_{rm}) &\sim Normal(0, 1.5) \\
& \quad \text{, for ventVT t = (8,6,4); ventRR r = (17,24,31); PPVmethod m = (gam, classic)}
\end{aligned}
$$

All independent variables are categorical. $PPVmethod$, m, is one of the categories “GAM” or “Classic”, $ventVT$, t, is one of the tidal volumes 10, 8, 6 or 4 ml kg^{-1} (10 ml kg^{-1} is the reference), $ventRR$, r, is one of the respiratory rates 10, 17, 24 or 31 min^{-1} (10 min^{-1} is the reference). We use categorical, rather than continuous, variables for tidal volume and respiratory rate, because we do not want to assume a linear effect of these settings, and because we want these model parameters to be directly interpretable as relative effects (after exponentiation). The random term (α_s) allows a subject specific intercept, reflecting that subjects present with PPVs in different ranges.

Model 2 (m2) is similar, but instead of separate effects of tidal volume ($ventVT$) and respiratory rate ($ventRR$), the two ventilator settings are combined to $ventSetting$, giving estimates of all 10 applied combinations of tidal volume and respiratory rate.

3.1 Priors

First we present the model priors. Generally these are weakly informative and only exclude unreasonably large effects. They simply serve as computational aids for fitting the model.

```r
# Population-level terms ---------------------------------------
# Because of the log-link, these terms represent the log of the
# multiplicative effect on the outcome scale.
priors_pterms <- c(
  # Prior for the default population level effects.
  # A normal distribution with SD = 2, means that any effect of ventilator settings
  # different than VT=10, RR=10 is probably (68% interval) between a 7x increase
  # and a 7x decrease in PPV. 95% prior interval exp(c(-4, 4) ~ 1/50 to 50.
  set_prior("normal(0, 2)", class = "b"),
  # Intercept (median PPV) is probably between 3 and 30 (i.e. exp(c(1.3, 3.3)) )
  # 95% prior interval ~ 1.4 to 73
  set_prior("normal(2.3, 1)", coef = "PPV_methodgam"),
  set_prior("normal(2.3, 1)", coef = "PPV_methodclassic")
)

# Variability terms ---------------------------------------
# Between-subject variability (random effect)
# and within-subject variability (residuals)
priors_ranef <- c(
  # Prior for sd of random effect (half-normal prior).
  # Since this effect is on the log scale, a sd of 1 would mean that
  # 68 % of subjects are within 2.7x below and above the value predicted from
  # the fixed effects.
  set_prior("normal(0,1.5)", class = "sd"),
  # Priors for the linear predictors of log(sigma): The residual variability.
  # This gives 68% prior probability that sigma at VT=10,RR=10 is in the
  # range exp(c(-1.5, 1.5)) = 0.22 to 4.48.
  # The relative effect of VT and RR on sigma is assumed to be less than 4.5x (each).
  set_prior("normal(0,1.5)", dpar = "sigma")
)

priors <- c(priors_pterms,
            priors_ranef)
```

3.2 Model sampling

The models are sampled using Stan, via the R interface `brms`. Four chains with 4000 post-warmup draws each were used.

```r
m1 <-
  brm(bf(PPV ~
           0 + PPV_method + (vent_rel_vt_f + vent_RR_f):PPV_method +
           (1 | id_f),
         sigma ~ 0 + PPV_method + (vent_rel_vt_f + vent_RR_f):PPV_method,
         # We fix nu (degrees of freedom in T distribution)
         nu = 4
  ),
```

```r
  prior = priors,
  data = PPV_df_long,
  seed = 1,
  iter = 6000,
  warmup = 2000,
  family = student(link = "log"),
  file = "temp_model_fits/m1",
  file_refit = "on_change")

# Model with interaction between VT and RR
m2 <-
  brm(bf(PPV ~
           0 + PPV_method + vent_setting:PPV_method +
           (1 | id_f),
         sigma ~ 0 + vent_setting:PPV_method,
         nu = 4
  ),
  prior = priors,
  data = PPV_df_long,
  seed = 1,
  iter = 6000,
  warmup = 2000,
  family = student(link = "log"),
  file = "temp_model_fits/m2",
  file_refit = "on_change")
```

4 Convergence

We consider that models have converged if `Rhat` for all parameters are < 1.01 (for details on the `Rhat` convergence measure, see Vehtari et al, 2021).

```r
rhat_m1 <- rhat(m1) |> na.omit() # when nu is fixed, Rhat for nu is NaN
rhat_m2 <- rhat(m2) |> na.omit()

stopifnot(max(rhat_m1) < 1.01)
stopifnot(max(rhat_m2) < 1.01)
```

m1: `max(rhat(m1))` $= 1.0028754$

m2: `max(rhat(m2))` $= 1.0027445$

5 Posterior predictive plots

Below are plots showing the posterior prediction of PPV for all 10 ventilator settings (8.10.gam means V_T=8 ml kg^{-1}, RR=10 min^{-1} with GAM method). The Student t distribution of the response places a (very) small area of the predictive distribution in negative PPV values. Negative

PPV values are not possible. We also fitted the models with a lower bound of 0 on the response distribution, eliminating negative predictions. That model gave essentially identical results, so we decided to use the non-bounded distribution, as it's location parameter (μ) is equal to the expected value, allowing interpretation of model parameters as conditional effects on the expected value of PPV.

```
bayesplot::ppc_dens_overlay_grouped(
  m1$data$PPV,
  yrep = posterior_epred(m1, ndraws = 50),
  group = with(m1$data, interaction(vent_rel_vt_f, vent_RR_f, PPV_method))) +
  labs(title = "m1 - Posterior predictive plots (VT.RR.method)",
       x = "PPV") +
  scale_x_continuous(limits = c(-5, 25))
```

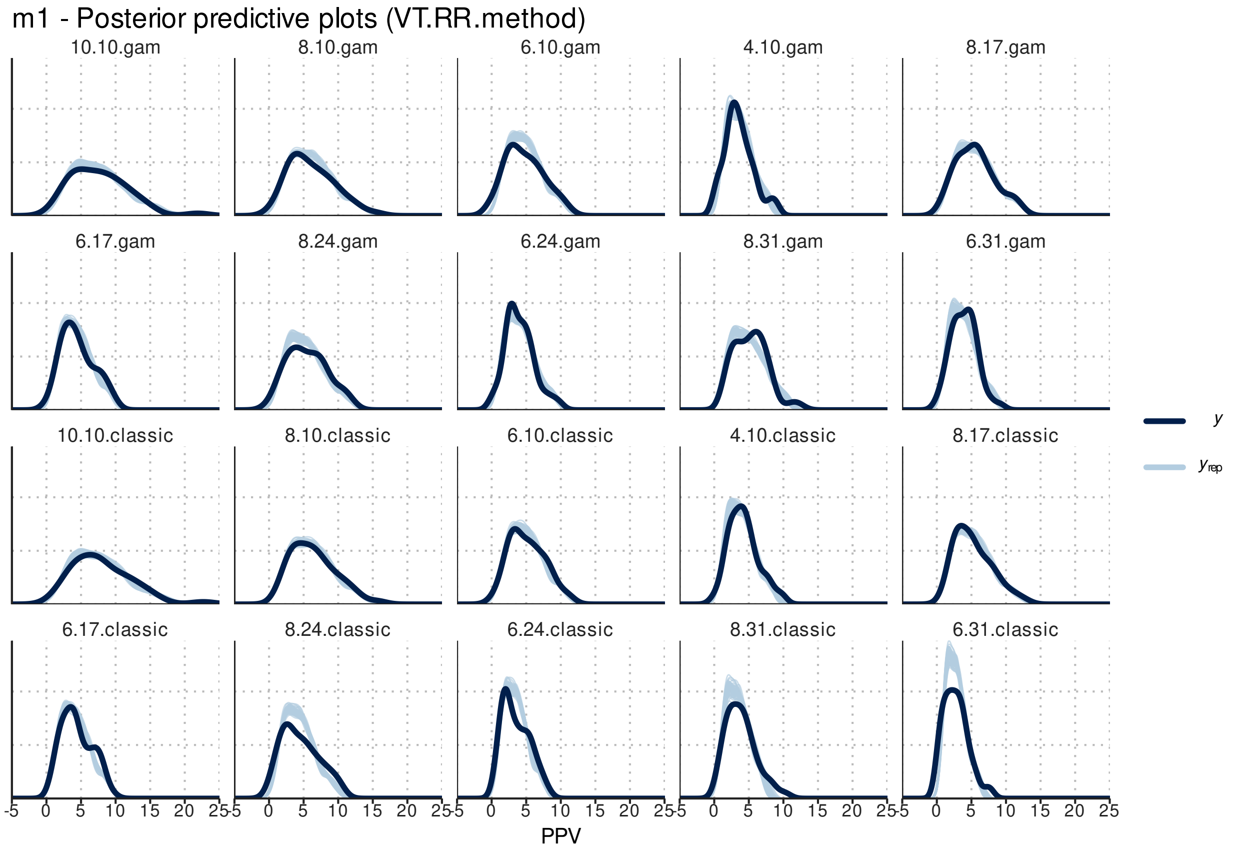

We only include the posterior predictive distributions for m1. The plots for m2 look practically identical.

6 Pareto K diagnostic and comparison of m1 and m2

```r
loo(m1, m2)
```

```
## Output of model 'm1':
##
## Computed from 16000 by 1014 log-likelihood matrix
##
##          Estimate   SE
## elpd_loo  -1548.5 33.7
## p_loo        91.4  3.2
## looic      3097.1 67.4
## ------
## Monte Carlo SE of elpd_loo is 0.1.
##
## All Pareto k estimates are good (k < 0.5).
## See help('pareto-k-diagnostic') for details.
##
## Output of model 'm2':
##
## Computed from 16000 by 1014 log-likelihood matrix
##
##          Estimate   SE
## elpd_loo  -1557.8 33.6
## p_loo       105.5  3.6
## looic      3115.6 67.1
## ------
## Monte Carlo SE of elpd_loo is 0.1.
##
## All Pareto k estimates are good (k < 0.5).
## See help('pareto-k-diagnostic') for details.
##
## Model comparisons:
##    elpd_diff se_diff
## m1  0.0       0.0
## m2 -9.3       3.4
```

All Pareto k are < 0.5 for both models, indicating that we do not have any overly influential data points. The higher elpd of model 1 indicates that this model is probably preferable (it is both simpler and performs better in cross-validation). In the paper we only consider model 1 as it is simpler to interpret. Here, we present both for completeness.

6.1 Variation in data explained by the model

```r
bayes_R2(m1)
```

```
##     Estimate   Est.Error      Q2.5     Q97.5
## R2 0.8345923 0.004773569 0.8245541 0.8433161
```

Model 1 explains ~83% of the variation in data.

```
bayes_R2(m1, re_formula = NA)
```

```
##     Estimate  Est.Error      Q2.5     Q97.5
## R2 0.1490064 0.02120653 0.1101846 0.1941463
```

If we exclude the random effects (between individual variation), we can see that the fixed effects explain ~15% of the variation. I.e. Within individuals, just shy of half the variation in PPV is explained by change in ventilator settings.

```
bayes_R2(m2)
```

```
##     Estimate   Est.Error      Q2.5     Q97.5
## R2 0.8355662 0.004910302 0.8252781 0.8445483
```

```
bayes_R2(m2, re_formula = NA)
```

```
##     Estimate  Est.Error      Q2.5     Q97.5
## R2 0.1516769 0.02085516 0.1141889 0.1950421
```

7 Make figure for m1 - No interaction

The following is the code to produce figure 5 in the paper.

7.1 Plot observed PPV

Plot of observed PPV for all ventilator settings and both methods (GAM and Classic)

```
observed_plot <- ggplot(PPV_df_long, aes(label, PPV)) +
  ggbeeswarm::geom_quasirandom(aes(color = PPV_method),
                               dodge.width=.6,
                               width = 0.1,
                               size = 0.7,
                               shape=16) +
  guides(color = guide_legend(override.aes = list(alpha = 1, size = 1))) +
  scale_color_discrete(limits = c("gam", "classic"), labels = c("GAM", "Classic")) +
  labs(title = "Observed data", x="", y="PPV [%]",
       color = "PPV method",
       tag = "a") +
  theme(axis.text.x = ggtext::element_markdown(hjust = 1, angle = 20),
        legend.position = c(0.5, 1),
        legend.direction = "horizontal",
        legend.justification = c(0.5,0.5),
```

```r
        legend.box.background = element_rect(color = NA, fill = "white"),
        legend.text = element_text(size = rel(1)))

observed_plot
```

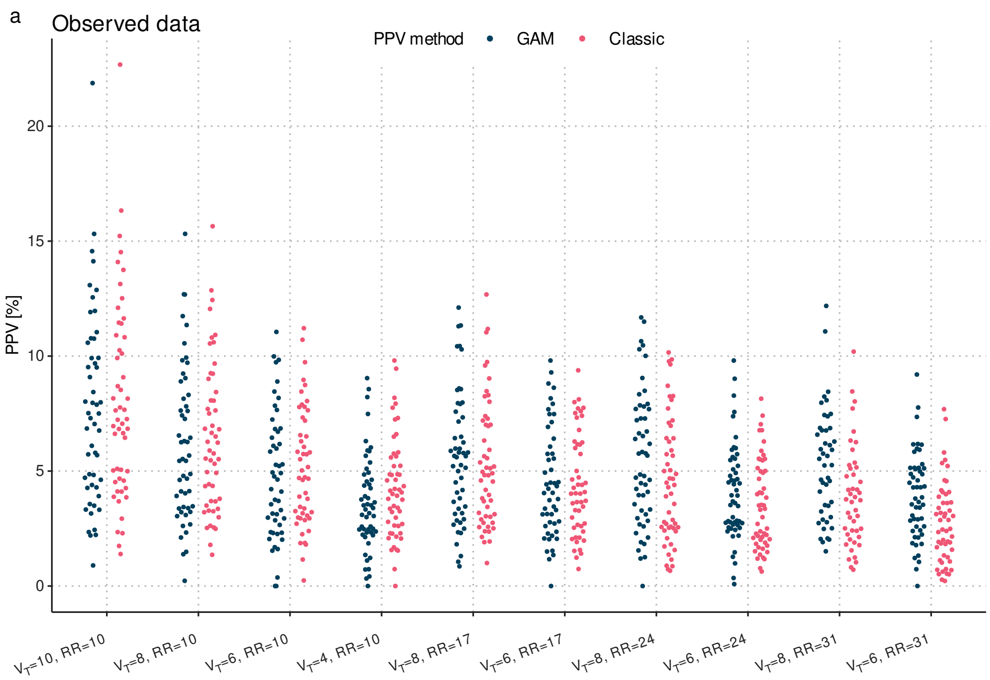

7.2 Plot ventilation effects

```r
# Intercepts
intercept_draws_m1 <- gather_draws(m1, `b_PPV_method(gam|classic)`, regex = TRUE) |>
  mutate(PPV_method = str_remove(.variable, "b_PPV_method") |>
           factor(levels = c("gam", "classic")),
         intercept = exp(.value),
         label = "V<sub>T</sub>=10, RR=10")

intercept_plot_m1 <- ggplot(intercept_draws_m1, aes(label, intercept, color = PPV_method)) +
  stat_pointinterval(point_size = 1,
                     interval_size = 1,
                     position = position_dodge(width = 0.4),
                     .width = 0.95) +
  coord_cartesian(ylim = c(0, 20)) +
  labs(x="", y="PPV", tag = "b",
       title = "Intercepts") +
  theme(legend.position = "none")

intercept_plot_m1
```

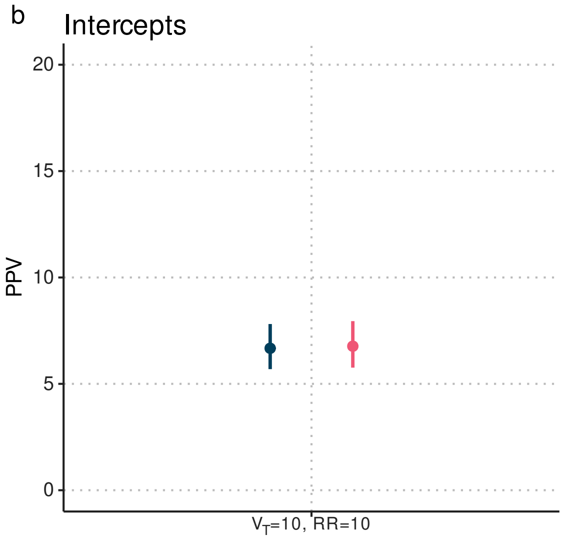


```r
# Contrasts
contrast_draws_m1 <- gather_draws(m1, `b_PPV_method(gam|classic):.+`, regex = TRUE) |>
  separate(.variable, into = c("PPV_method", "setting"), sep = ":") |>
  separate(setting, into = c("setting_type", "setting"), sep = "_f") |>
  mutate(PPV_method = str_remove(PPV_method, "b_PPV_method") |>
           factor(levels = c("gam", "classic")),
         rel_effect = exp(.value))

contrast_draws_vt_m1 <- filter(contrast_draws_m1, setting_type == "vent_rel_vt") |>
  mutate(label = vt_levels[setting] |> factor(levels = vt_levels))

contrast_plot_layers <- list(
  stat_pointinterval(point_size = 1,
                     interval_size = 1,
                     position = position_dodge(width = 0.4),
                     .width = 0.95),
  labs(y = "Relative effect", x = ""),
  scale_y_continuous(labels = scales::label_percent(accuracy = 1),
                     breaks = seq(0.4, 1, by = 0.2)),
  coord_cartesian(ylim = c(0.4, 1)),
  theme(legend.position = "none")
)

contrast_plot_vt_m1 <- ggplot(contrast_draws_vt_m1,
                              aes(label, rel_effect, color = PPV_method)) +
  contrast_plot_layers +
  labs(title = "Effect of tidal volume (V<sub>T</sub>) on PPV",
       subtitle = "Relative to V<sub>T</sub>=10 ml kg⁻¹", tag = "c")
```

```r
contrast_draws_rr_m1 <- filter(contrast_draws_m1, setting_type == "vent_RR") |>
  mutate(label = rr_levels[setting] |> factor(levels = rr_levels))

contrast_plot_rr_m1 <- ggplot(contrast_draws_rr_m1,
                              aes(label, rel_effect, color = PPV_method)) +
  contrast_plot_layers +
  labs(title = "Effect of respiratory rate (RR) on PPV",
       subtitle = "Relative to RR=10 min⁻¹", tag = "d")


contrast_plot_vt_m1 + contrast_plot_rr_m1
```

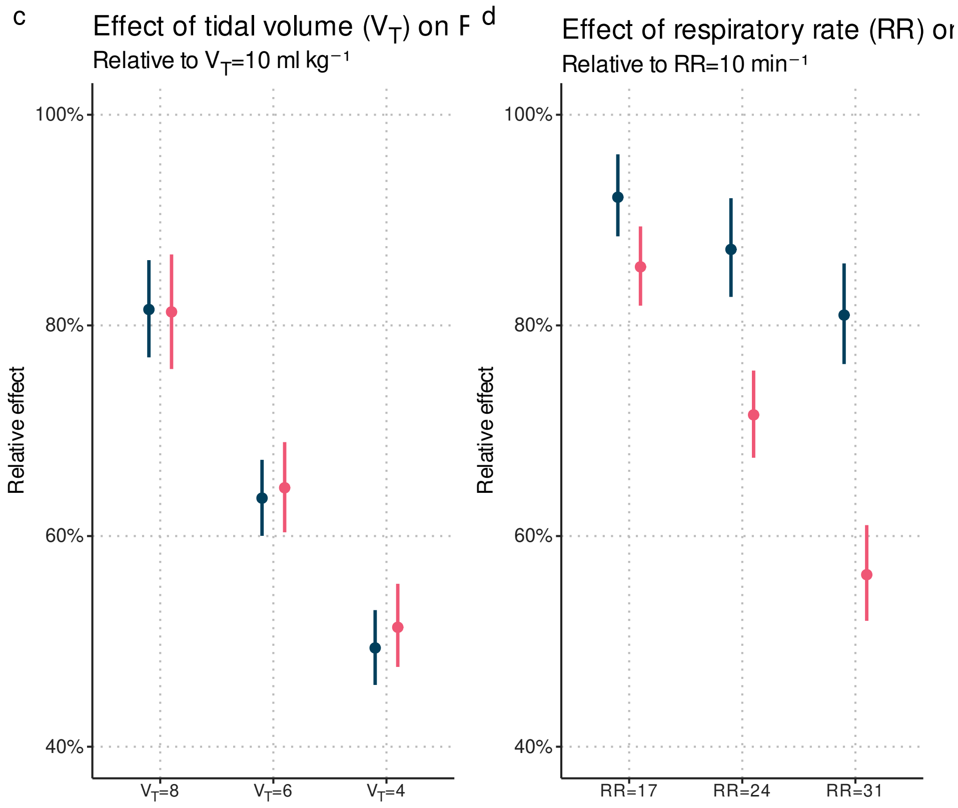

7.3 Make figure 5 (without CV and residuals)

```r
param_plot_design_m1_simple <- "
  AAA
  BCD
"

m1_plot_simple <- observed_plot +
```

```r
  intercept_plot_m1 + contrast_plot_vt_m1 + contrast_plot_rr_m1 +
  plot_layout(design = param_plot_design_m1_simple,
              heights = c(2, 3),
              widths = c(1, 3, 3)
              )

save_plot("fig6_mix_model_fig", m1_plot_simple, width = 18, height = 11, scale = 1)
```

```
## [1] "plots/fig6_mix_model_fig.png" "plots/fig6_mix_model_fig.pdf"
```

7.4 Plot residuals

```r
# Get mean residuals for both m1 and m2.
PPV_df_long_resid <- PPV_df_long |>
  mutate(
    resid_m1 = residuals(m1, method = "posterior_predict")[,"Estimate"],
    resid_m2 = residuals(m2, method = "posterior_predict")[,"Estimate"]
  )

resid_plot_m1 <-  ggplot(PPV_df_long_resid,
                         aes(label, resid_m1, color = PPV_method)) +
  ggbeeswarm::geom_quasirandom(dodge.width=.6,
                               width = 0.1,
                               size = 0.5,
                               shape=16) +
  stat_summary(aes(color = NULL, group = PPV_method), fun = mean, geom = "point",
               shape = "-", size = 5,
               position = position_dodge(width = 0.6)) +
  labs(title = "Model residuals (observed PPV - expected PPV)",
       subtitle = "The horizontal segments are the means of the residuals",
       x="", y="Residual",
       tag = "e") +
  theme(axis.text.x = ggtext::element_markdown(hjust = 1, angle = 20),
        legend.position = "none")

resid_plot_m1
```

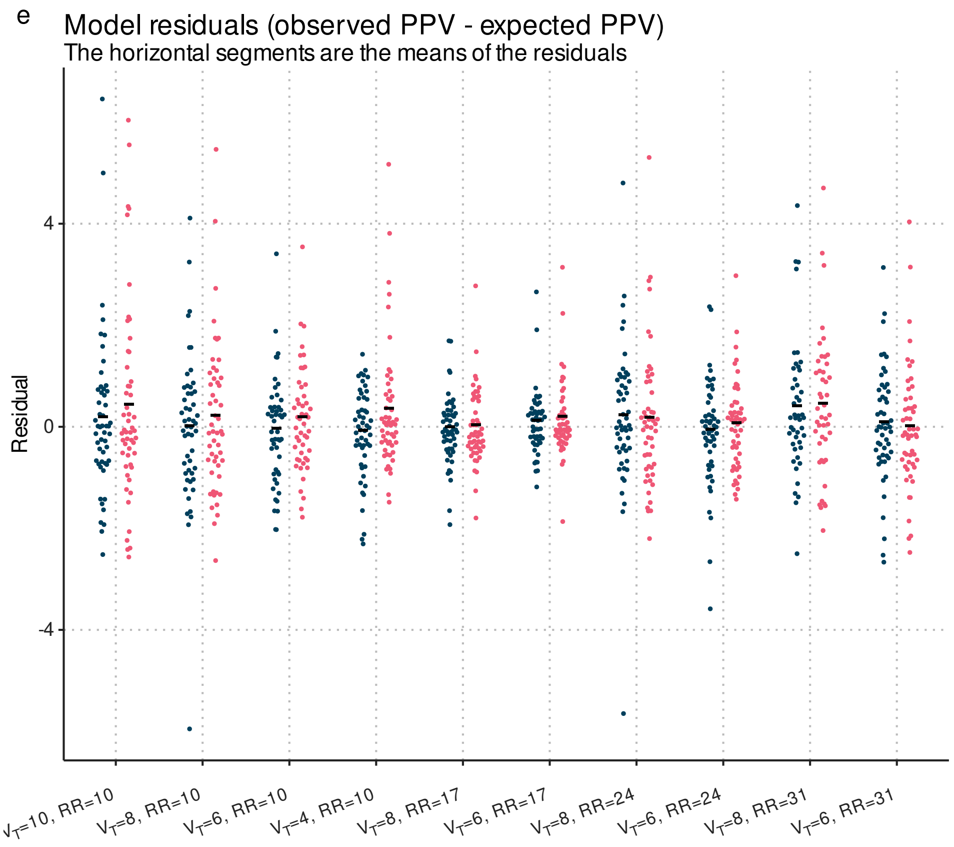

7.5 Plot residual standard deviation

Since we use a student t distribution for the likelihood, the sigma parameter does not equal the standard deviation (SD). SD of a T distribution is

$$SD = \sqrt{\sigma^2 \frac{\nu}{\nu - 2}}, for\ \nu > 2$$

where ν (nu) is the degrees of freedom parameter.

```
sd_t <- function(sigma, nu) {
  stopifnot(nu > 2)
  sqrt( sigma^2 * (nu / (nu-2)) )
}
```

```
# Make a data frame with one row for each combination of PPV_method and vent_setting
newdata_method_setting <- PPV_df_long |>
  tidyr::expand(PPV_method, nesting(vent_setting,
                                    vent_rel_vt, vent_RR,
                                    vent_rel_vt_f, vent_RR_f))

# Make draws of mean of posterior predictions (epred)
```

```r
# include sigma and nu for each draw (they are used to calculate SD).
vent_setting_epred_m1 <- newdata_method_setting |>
  add_epred_draws(m1, re_formula = NA,
                  dpar = c("sigma", "nu")) |>
  mutate(label = vent_setting_levels[as.character(vent_setting)] |>
           factor(levels = vent_setting_levels),
         SD = sd_t(sigma, nu),
         CV = SD/.epred)

sd_plot_m1 <- ggplot(vent_setting_epred_m1, aes(label,
                                               CV,
                                               color = PPV_method)) +
  stat_pointinterval(position = position_dodge(width = 0.3),
                     .width = 0.95, interval_size = 1,
                     point_size = 1, show.legend = FALSE) +
  scale_y_continuous(limits = c(0, NA), labels = scales::label_percent()) +
  labs(title = "Residual coefficient of variation [CV = SD(residuals) / E(PPV)]",
       x="", y="CV", color = "PPV method",
       tag = "f") +
  theme(axis.text.x = ggtext::element_markdown(hjust = 1, angle = 20))

sd_plot_m1
```

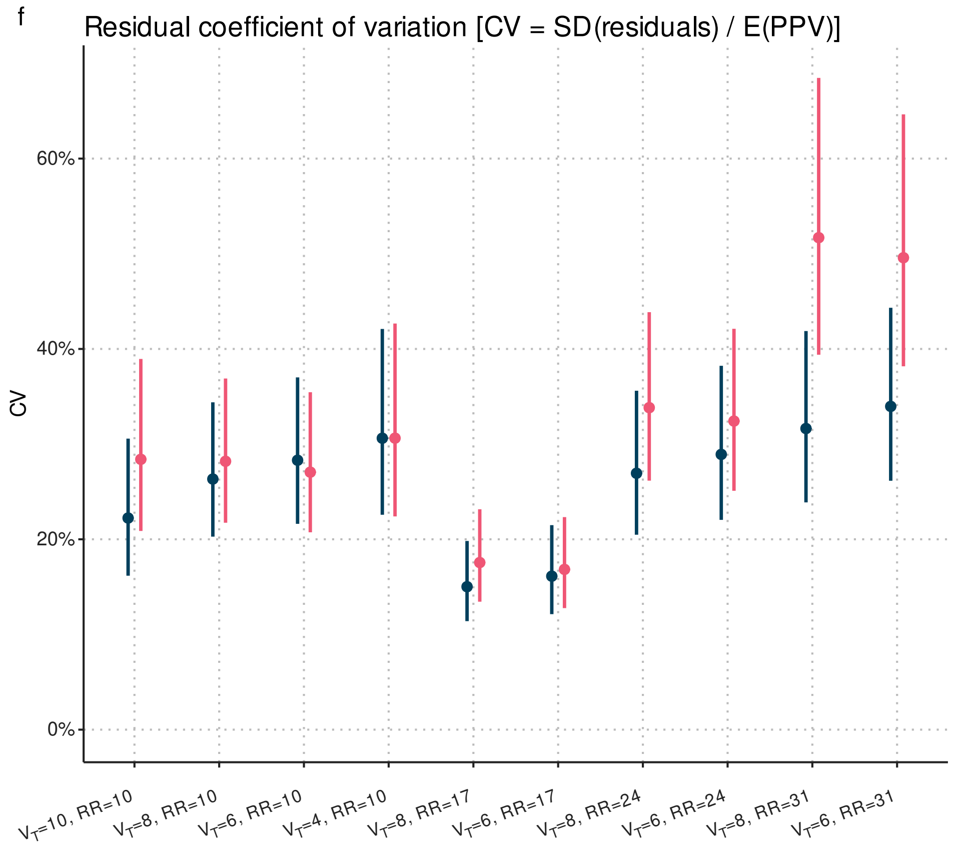

7.6 Combine plots in one figure

```
param_plot_design_m1 <- "
  AAA
  BCD
  EEE
  FFF
"

m1_plot <- observed_plot +
  intercept_plot_m1 + contrast_plot_vt_m1 + contrast_plot_rr_m1 +
  resid_plot_m1 +
  sd_plot_m1 +
  plot_layout(design = param_plot_design_m1,
              heights = c(1, 1.5, 1, 1),
              widths = c(1, 3, 3)
              )

save_plot("suppl_m1_plot", m1_plot, width = 18, height = 18, scale = 1)
```

```
## [1] "plots/suppl_m1_plot.png" "plots/suppl_m1_plot.pdf"
```

8 Make table of relative effects for m1 (relative to V_T=10 ml kg^{-1}, RR=10 min^{-1})

These are the estimates that are visualized in panel c and d.

```
contrast_draws_m1 |>
  group_by(PPV_method, setting_type, setting) |>
  median_qi(rel_effect) |>
  mutate(label = sprintf("%.0f [%.0f; %.0f]%%",
                         rel_effect * 100,
                         .lower * 100,
                         .upper * 100)) |>
  select(-c(.width, .point, .interval)) |>
  knitr::kable(booktabs = TRUE, digits = 2)
```

PPV_method	setting_type	setting	rel_effect	.lower	.upper	label
gam	vent_RR	17	0.92	0.88	0.96	92 [88; 96]%
gam	vent_RR	24	0.87	0.83	0.92	87 [83; 92]%
gam	vent_RR	31	0.81	0.76	0.86	81 [76; 86]%
gam	vent_rel_vt	4	0.49	0.46	0.53	49 [46; 53]%
gam	vent_rel_vt	6	0.64	0.60	0.67	64 [60; 67]%
gam	vent_rel_vt	8	0.82	0.77	0.86	82 [77; 86]%
classic	vent_RR	17	0.86	0.82	0.89	86 [82; 89]%
classic	vent_RR	24	0.72	0.67	0.76	72 [67; 76]%
classic	vent_RR	31	0.56	0.52	0.61	56 [52; 61]%
classic	vent_rel_vt	4	0.51	0.48	0.55	51 [48; 55]%
classic	vent_rel_vt	6	0.65	0.60	0.69	65 [60; 69]%
classic	vent_rel_vt	8	0.81	0.76	0.87	81 [76; 87]%

9 Compare m1 coefficient of variation (CV) between $PPV_{Classic}$ and PPV_{GAM} across ventilator settings

```
vent_setting_epred_m1 |>
  ungroup() |>
  pivot_wider(id_cols =c(.draw, vent_setting),
              names_from = PPV_method, values_from = CV, names_prefix = "CV_") |>
  mutate(CV_classic_m_gam = CV_classic - CV_gam) |>
  group_by(vent_setting) |>
  select(vent_setting, CV_classic_m_gam) |>
  median_qi(CV_classic_m_gam) |>
  mutate(label = sprintf("%.0f [%.0f; %.0f]%%-points",
                         CV_classic_m_gam * 100,
                         .lower * 100,
                         .upper * 100)) |>
  select(-c(.width, .point, .interval)) |>
  knitr::kable(booktabs = TRUE, digits = 2)
```

vent_setting	CV_classic_m_gam	.lower	.upper	label
10.10	0.06	-0.03	0.17	6 [-3; 17]%-points
8.10	0.02	-0.06	0.10	2 [-6; 10]%-points
6.10	-0.01	-0.10	0.07	-1 [-10; 7]%-points
4.10	0.00	-0.12	0.13	-0 [-12; 13]%-points
8.17	0.03	-0.03	0.08	3 [-3; 8]%-points
6.17	0.01	-0.05	0.06	1 [-5; 6]%-points
8.24	0.07	-0.02	0.17	7 [-2; 17]%-points
6.24	0.03	-0.06	0.13	3 [-6; 13]%-points
8.31	0.20	0.07	0.36	20 [7; 36]%-points
6.31	0.15	0.03	0.30	15 [3; 30]%-points

10 Make figure for m2 - Model that allows interaction of V_T and RR effects

10.1 Plot ventilation effects

```
intercept_draws_m2 <- gather_draws(m2, `b_PPV_method(gam|classic)`, regex = TRUE) |>
  mutate(PPV_method = str_remove(.variable, "b_PPV_method") |>
           factor(levels = c("gam", "classic")),
         intercept = exp(.value),
         label = "V<sub>T</sub>=10, RR=10")

intercept_plot_m2 <- intercept_plot_m1 %+% intercept_draws_m2

intercept_plot_m2
```

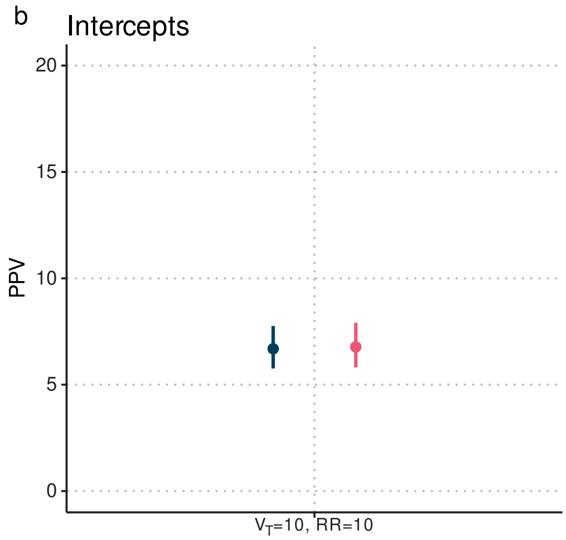


```
contrast_draws_m2 <- gather_draws(m2, `b_PPV_method(gam|classic):.+`, regex = TRUE) |>
  separate(.variable, into = c("PPV_method", "vent_setting"), sep = ":") |>
  mutate(PPV_method = str_remove(PPV_method, "b_PPV_method") |>
           factor(levels = c("gam", "classic")),
         vent_setting = str_remove(vent_setting, "vent_setting"),
         label = vent_setting_levels[vent_setting] |>
           factor(levels = vent_setting_levels),
         rel_effect = exp(.value))

contrast_plot_m2 <- ggplot(contrast_draws_m2,
                           aes(label, rel_effect, color = PPV_method)) +
  stat_pointinterval(point_size = 1,
                     interval_size = 1,
                     position = position_dodge(width = 0.4),
```

```r
                      .width = 0.95) +
  labs(y = "Relative effect", x = "",
       title = "Effect of tidal volume (V<sub>T</sub>) and respiratory rate (RR) on PPV",
       subtitle = "Relative to V<sub>T</sub>=10 ml kg⁻¹ and RR = 10 min⁻¹", tag = "c") +
  scale_y_continuous(labels = scales::label_percent(accuracy = 1),
                      breaks = seq(0.4, 1, by = 0.2)) +
  coord_cartesian(ylim = c(0.3, 1)) +
  theme(legend.position = "none",
        axis.text.x = ggtext::element_markdown(hjust = 1, angle = 20))


contrast_plot_m2
```

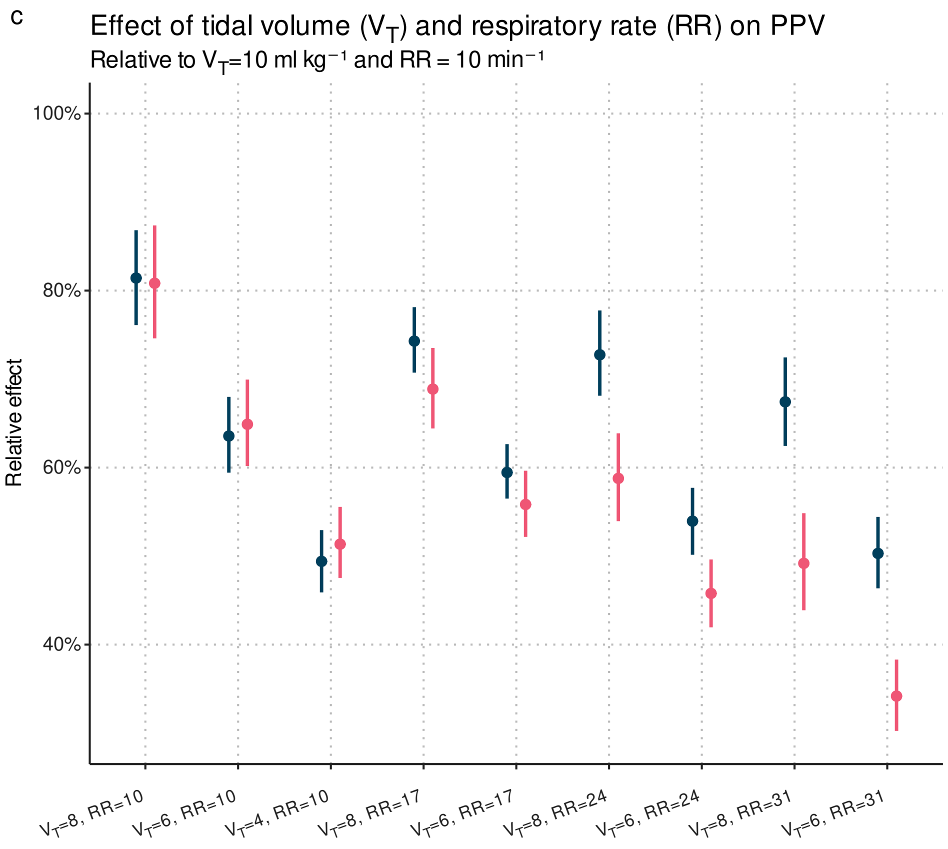

10.2 Plot residuals

```r
resid_plot_m2 <-  ggplot(PPV_df_long_resid, aes(label, resid_m2)) +
  ggbeeswarm::geom_quasirandom(aes(color = PPV_method),
                               dodge.width=.6,
                               width = 0.1,
                               size = 0.7,
```

```
                                   shape=16) +
  stat_summary(aes(color = NULL, group = PPV_method), fun = mean, geom = "point",
               shape = "-", size = 5,
               position = position_dodge(width = 0.6)) +
  labs(title = "Model residuals (observed PPV - expected PPV)",
       subtitle = "The horizontal segments are the means of the residuals",
       x="", y="Residual",
       tag = "d") +
  theme(axis.text.x = ggtext::element_markdown(hjust = 1, angle = 20),
        legend.position = "none")

resid_plot_m2
```

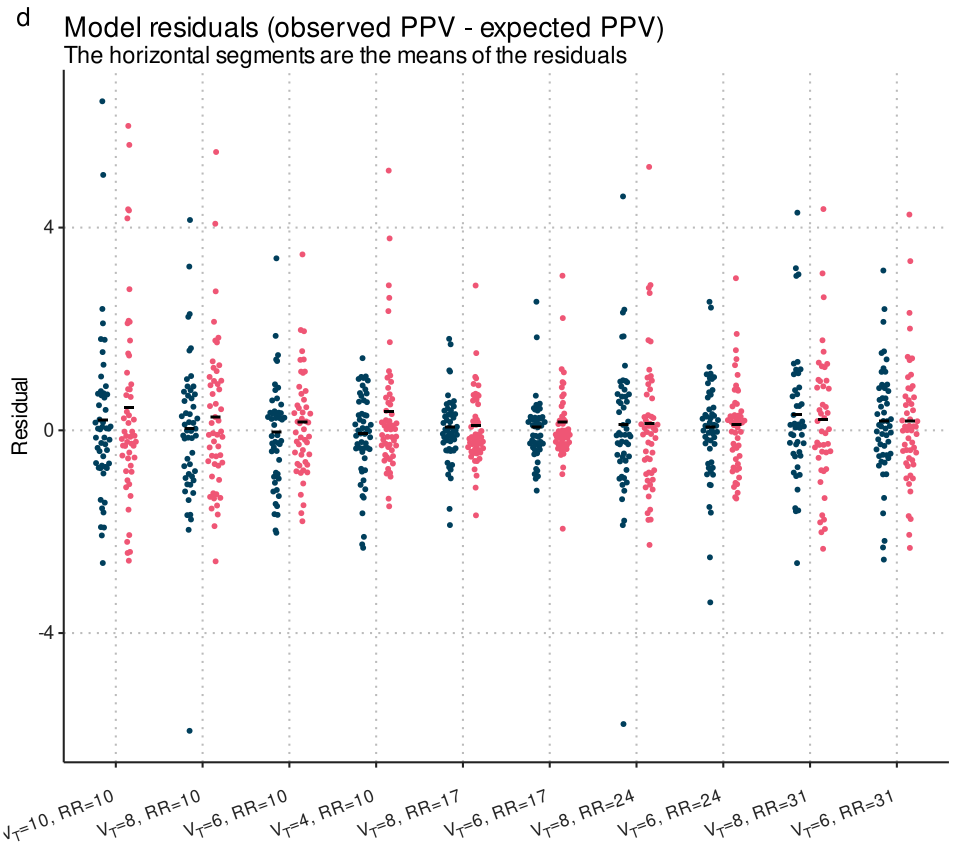

10.3 Plot residual standard deviation

```
# Make draws of mean of posterior predictions (epred)
# include sigma for each draw.

vent_setting_epred_m2 <- newdata_method_setting |>
  add_epred_draws(m2, re_formula = NA,
```

```
                   dpar = c("sigma", "nu")) |>
  mutate(label = vent_setting_levels[as.character(vent_setting)] |>
           factor(levels = vent_setting_levels),
         SD = sd_t(sigma, nu),
         CV = SD/.epred)

# Reuse the sd plot from model 1, but with new data
sd_plot_m2 <- sd_plot_m1 %+% vent_setting_epred_m2 +
  labs(tag = "e")

sd_plot_m2
```

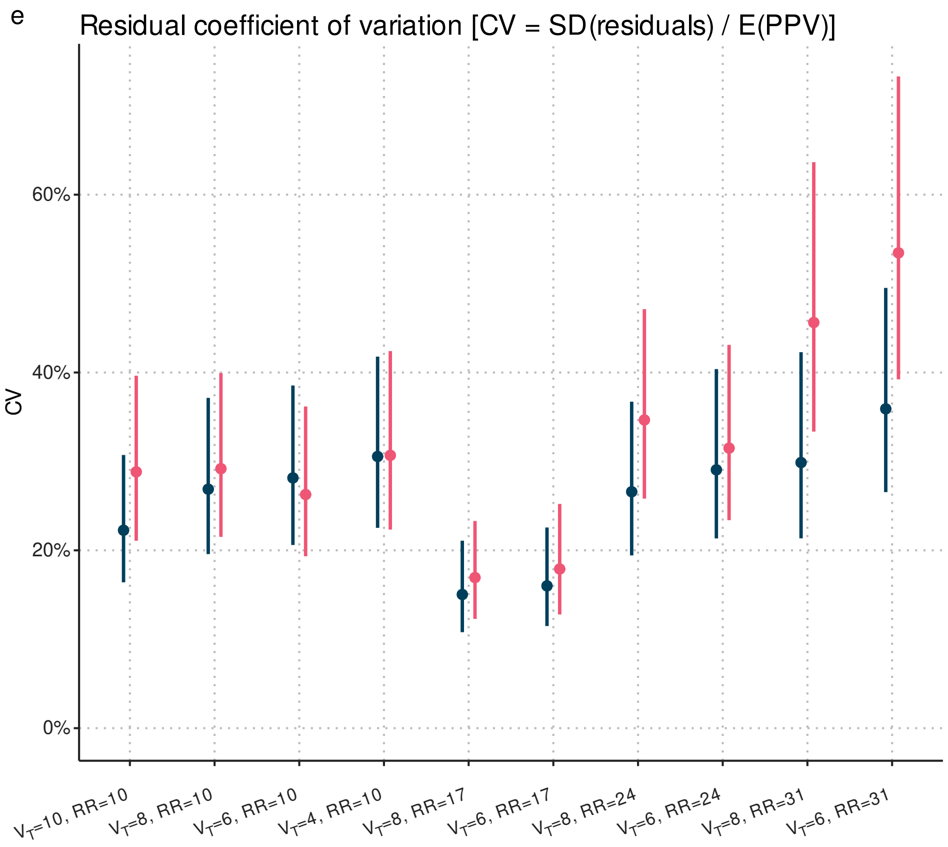

10.4 Combine plots in one figure

```
param_plot_design_m2 <- "
  AA
  BC
  DD
  EE
"
```

```r
m2_plot <- observed_plot +
  intercept_plot_m2 +
  contrast_plot_m2 +
  resid_plot_m2 +
  sd_plot_m2 +
  plot_layout(design = param_plot_design_m2,
              heights = c(1, 1.5, 1, 1),
              widths = c(1, 5)
              )

save_plot("extra_m2_plot", m2_plot, width = 18, height = 18, scale = 1)
```

```
## [1] "plots/extra_m2_plot.png" "plots/extra_m2_plot.pdf"
```

```r
m2_plot
```

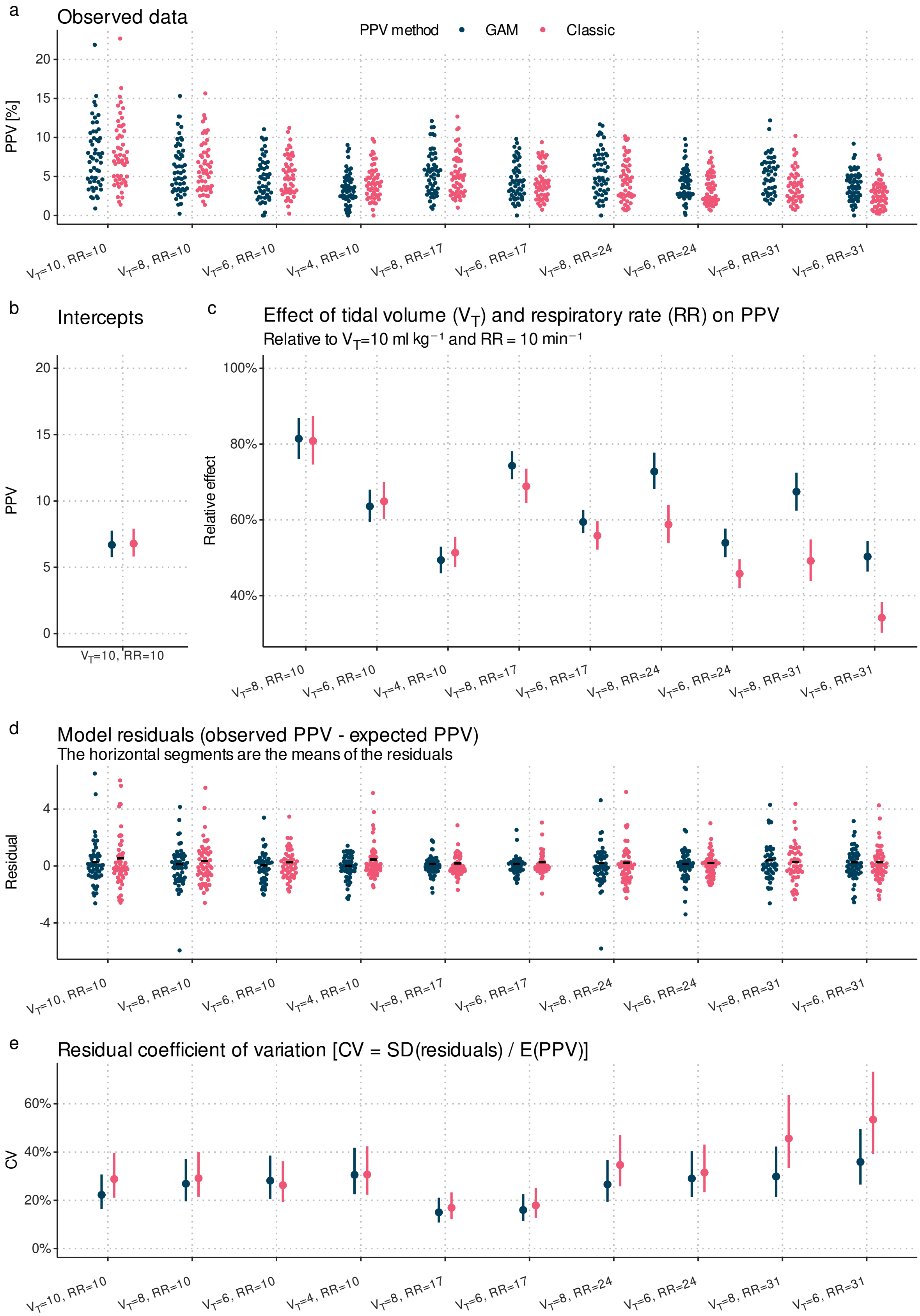

a
Observed data
PPV method GAM Classic
PPV [%]
V_T=10, RR=10
V_T=8, RR=10
V_T=6, RR=10
V_T=4, RR=10
V_T=8, RR=17
V_T=6, RR=17
V_T=8, RR=24
V_T=6, RR=24
V_T=8, RR=31
V_T=6, RR=31
b
Intercepts
PPV
c
Effect of tidal volume (V_T) and respiratory rate (RR) on PPV
Relative to V_T=10 ml kg^{-1} and RR = 10 min^{-1}
Relative effect
d
Model residuals (observed PPV - expected PPV)
The horizontal segments are the means of the residuals
Residual
e
Residual coefficient of variation [CV = SD(residuals) / E(PPV)]
CV
