# Supplementary material for: The effects of respiratory rate and tidal volume on pulse pressure variation in healthy lungs–a generalized additive model approach may help overcome limitations
Source: J Clin Monit Comput. 2023 Nov 16;38(1):57–67. doi: 10.1007/s10877-023-01090-6 (PMC10879304; doi:10.1007/s10877-023-01090-6)

Overview of the beats used to calculate standard PPV.
Excluded beats are also excluded in the GAM PPV calculation.
The smooth line is the trending part of the GAM PPV calculation.
The corresponeding cyclic effects part of the GAM is shown on the next page.

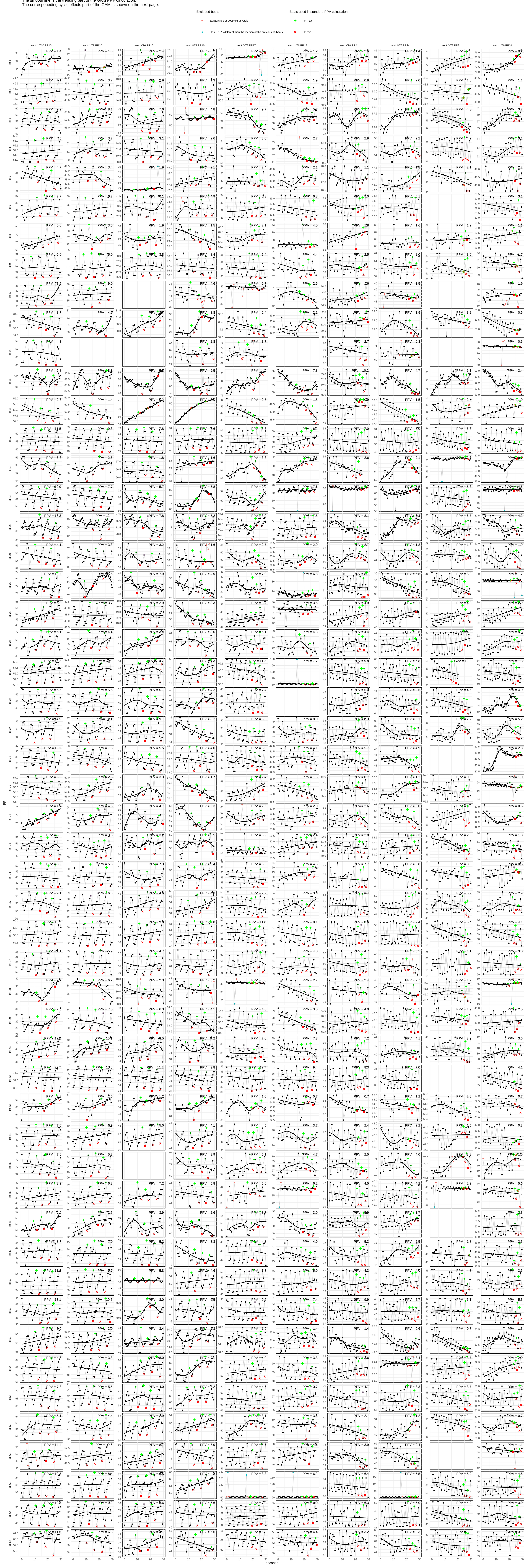

Pulse pressure through respiratory cycle.
The peak-to-peak amplitude divided by the mean PP is the GAM PPV.

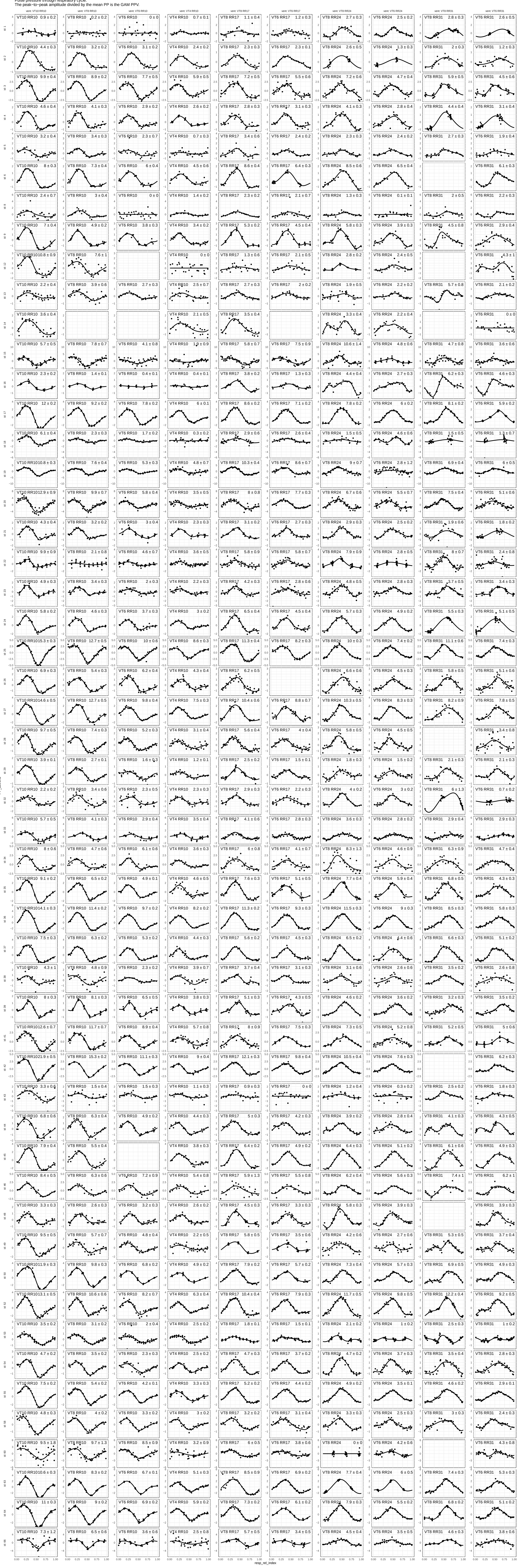

Supplement: Supplementary file 2 — Supplementary file2 (PDF 2678 KB) [file 10877_2023_1090_MOESM2_ESM.pdf]
