# Supplementary material for: The effects of respiratory rate and tidal volume on pulse pressure variation in healthy lungs–a generalized additive model approach may help overcome limitations
Source: J Clin Monit Comput. 2023 Nov 16;38(1):57–67. doi: 10.1007/s10877-023-01090-6 (PMC10879304; doi:10.1007/s10877-023-01090-6)
Supplement: Supplementary file 3 — Supplementary file3 (PDF 237 KB) [file 10877_2023_1090_MOESM3_ESM.pdf]

Supplementary figures and tables

Figure S1

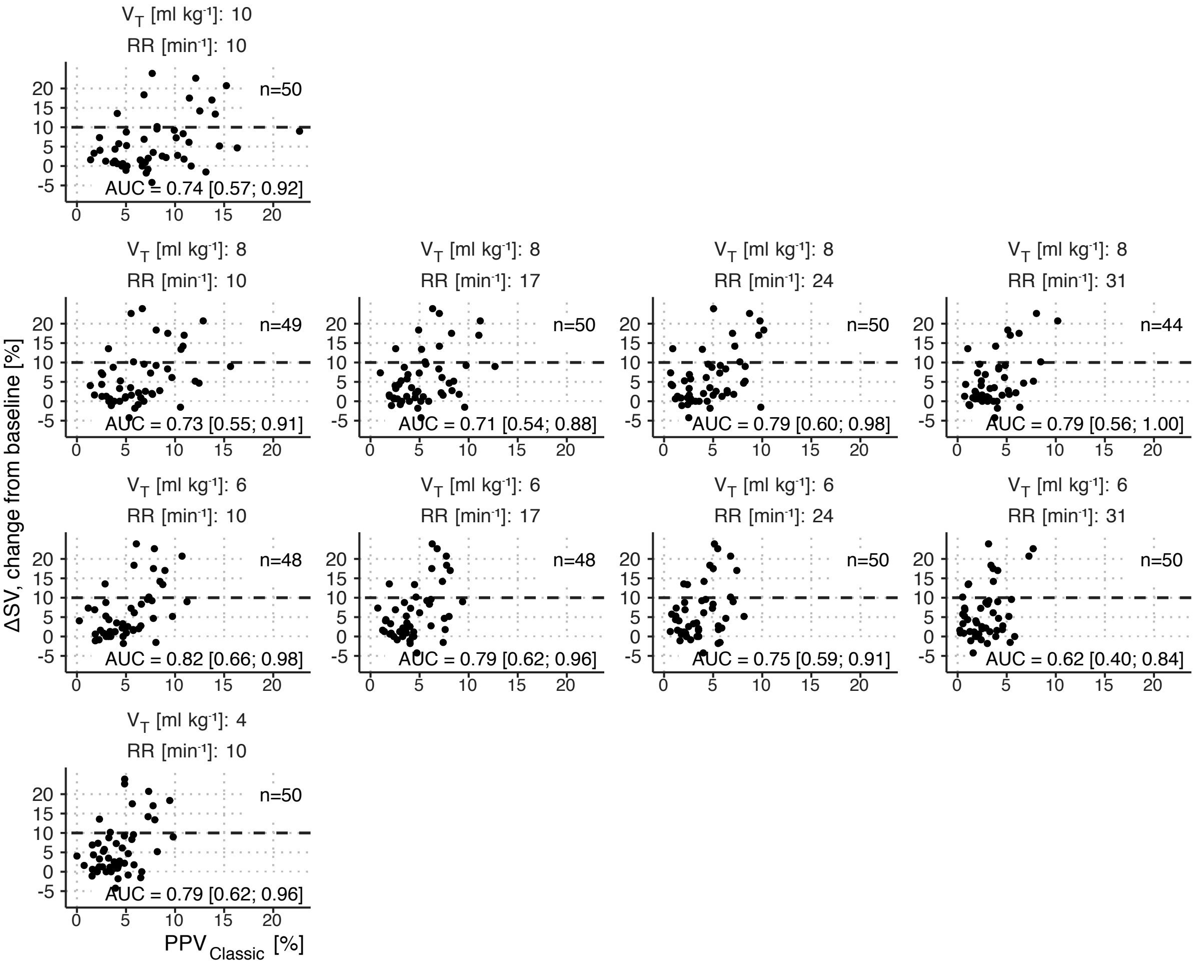

Figure S1: Scatter plots of the relation between PPV calculated with the classic algorithm ($\text{PPV}_{\text{Classic}}$) and the stroke volume response (ΔSV) to a 250 ml fluid challenge. Panels are arranged with tidal volumes (V_T) in rows and respiratory rates (RR) in columns . One fluid challenge was evaluated for each subject (n = 50), while $\text{PPV}_{\text{Classic}}$ was calculated for each of the 10 ventilator settings.

Figure S2

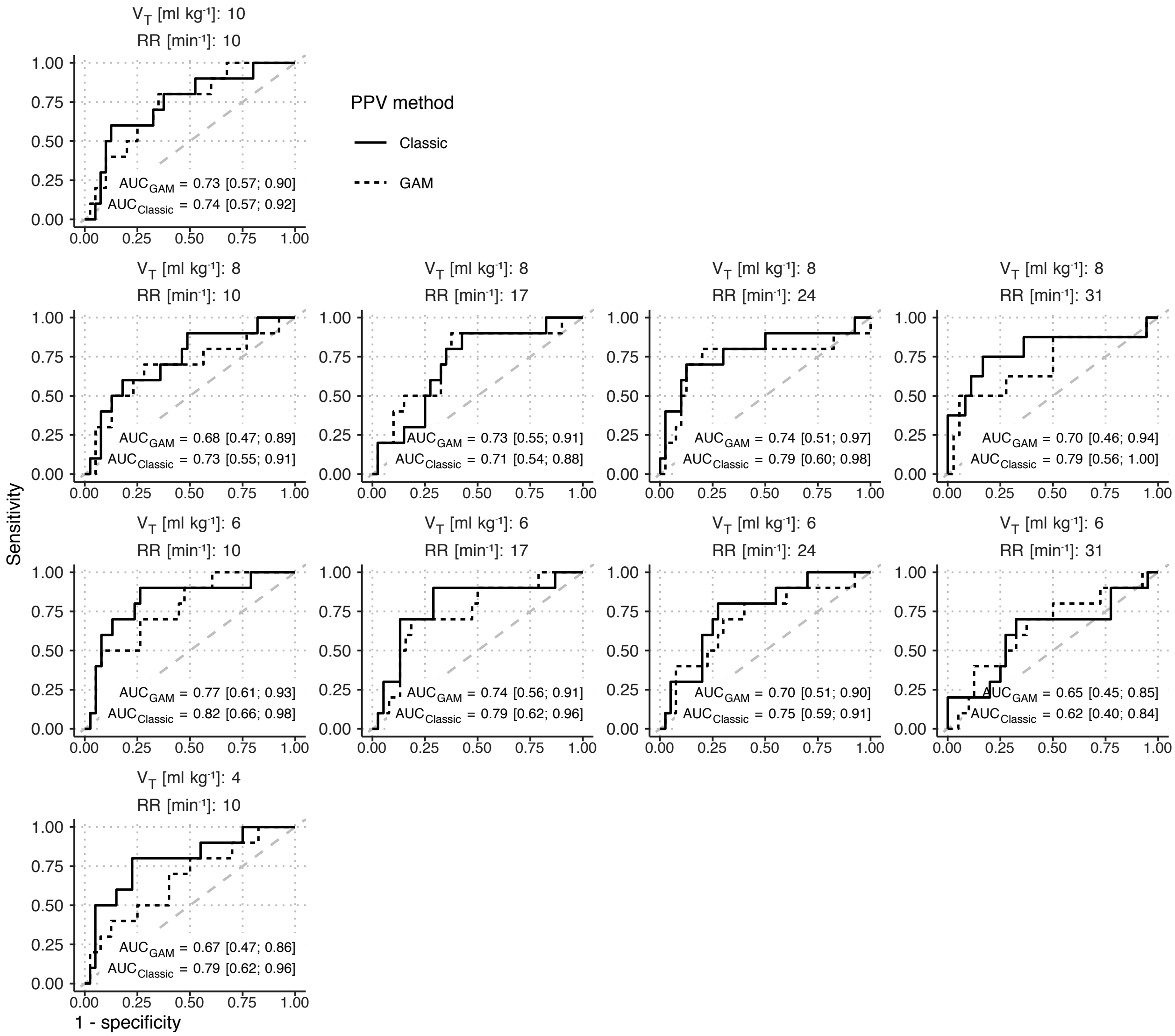

Figure S2: Receiver operating characteristic (ROC) curves for capacity of pulse pressure variation (PPV) to classify fluid responsiveness (stroke volume change > 10%). Panels are arranged with tidal volumes (V_T) in rows and respiratory rates (RR) in columns. One fluid challenge was evaluated for each subject (n = 50), while PPV_{GAM} and $PPV_{Classic}$ was calculated for each of the 10 ventilator settings.

Figure S3

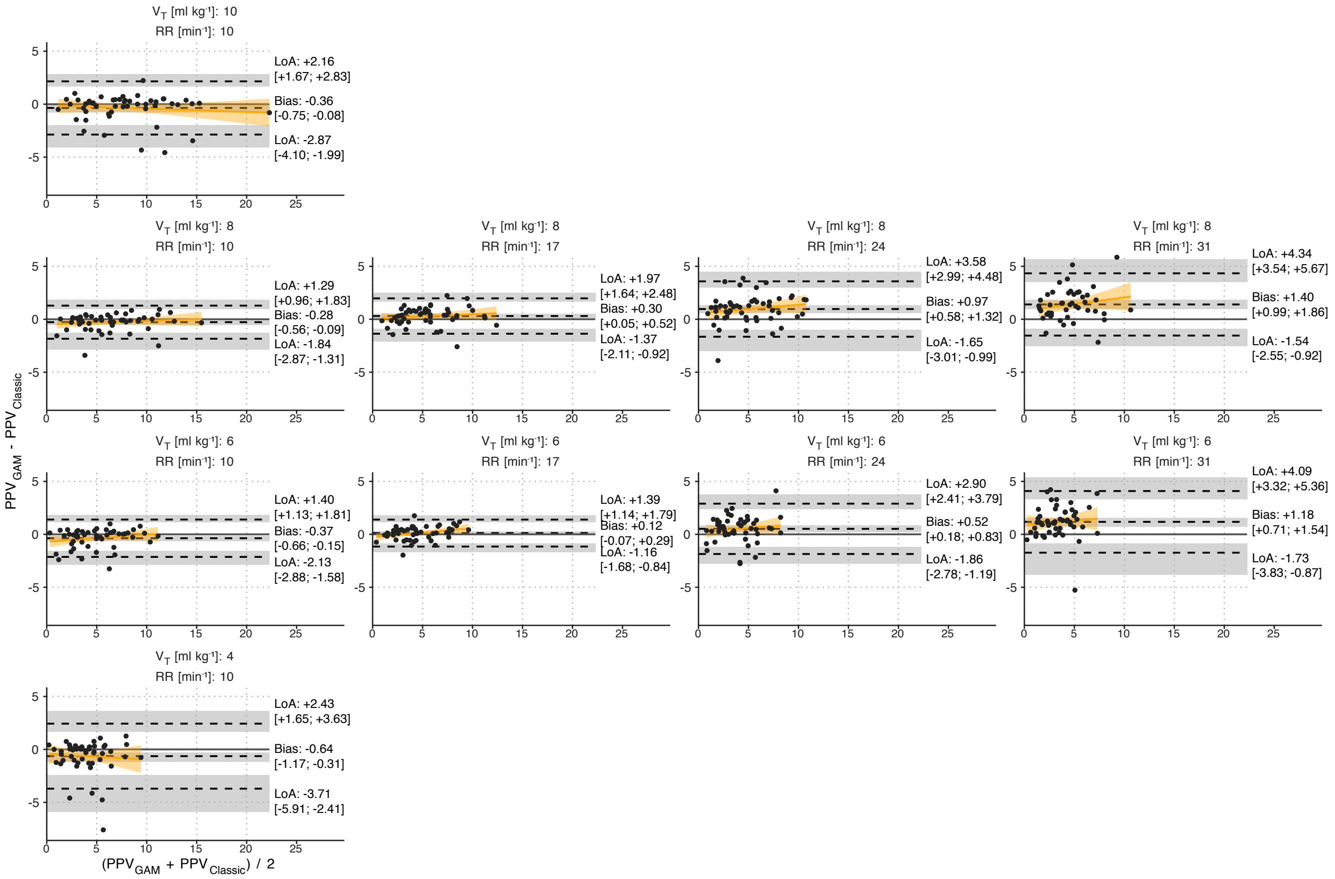

Figure S3: Bland-Altman plots showing the relation between PPV_{GAM} and $PPV_{Classic}$. The outer dashed lines represent 95% limits of agreement (LoA). Grey areas are 95% confidence intervals for bias and LoA. The purple lines and areas are linear regression fits with 95% confidence intervals. Panels are arranged with tidal volumes (V_T) in rows and respiratory rates (RR) in columns.

Figure S4

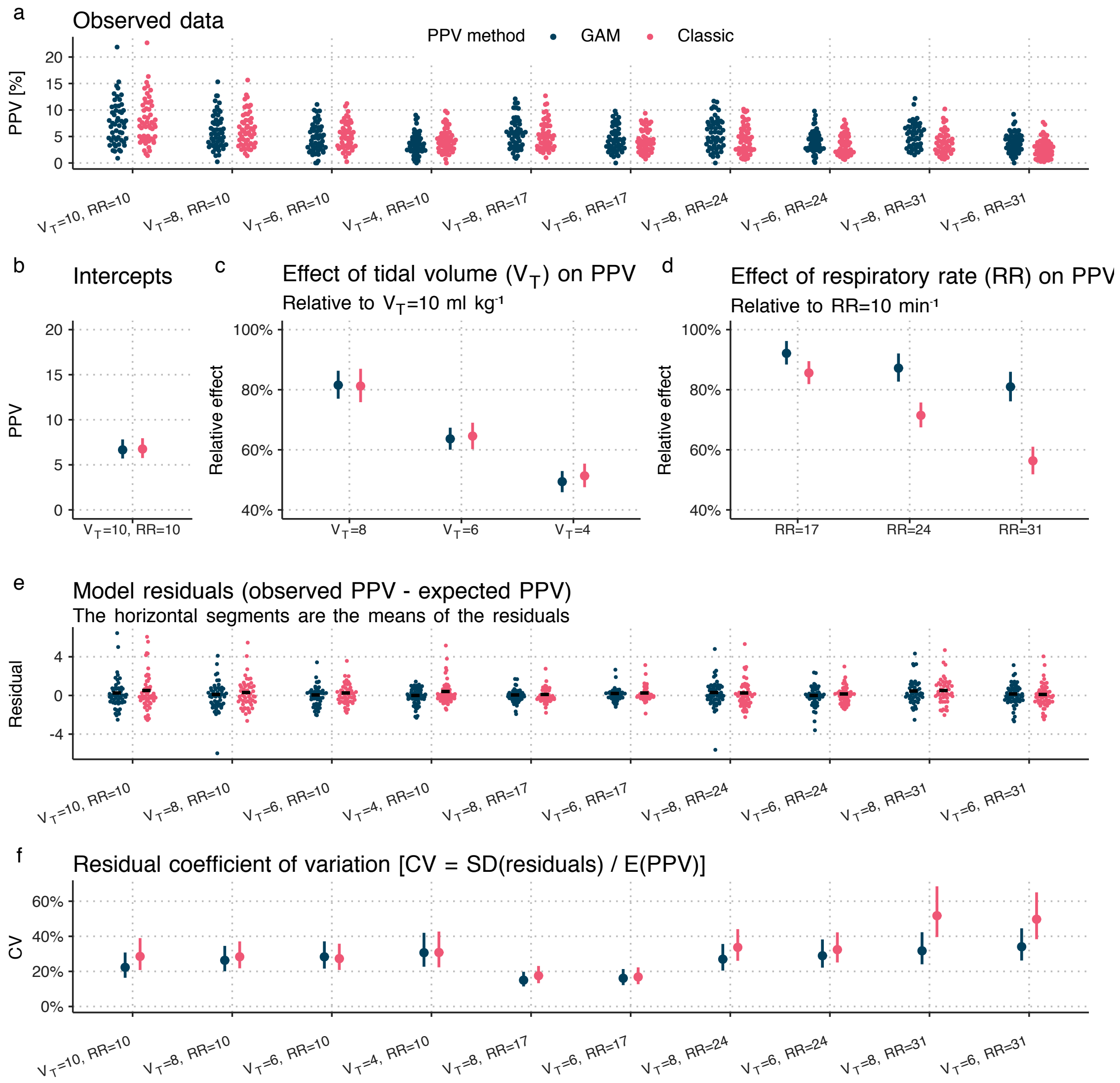

Figure S4: (Extended version of the paper's Fig. 5): Parameter estimates for a Bayesian mixed-effects model, describing the effect of tidal volume (V_T) and respiratory rate (RR) on pulse pressure variation (PPV). Parameters are estimated for both PPV derived using a generalized additive model (GAM) and using a classic approach (Classic). Panel **a** presents the observed PPV values (outcomes) using each method (n=507 for both GAM and Classic). Vertical bars are 95% compatibility intervals. Panel **b**, **c** and **d** present parameter estimates. Vertical bars are 95% compatibility intervals. Panel **e** shows model residuals, and panel **f** shows the residual variation relative to the estimated value of PPV.

Table S1

Receiver operating characteristic analysis of fluid-responsiveness prediction using pulse pressure variation (PPV). A positive fluid response was a >10% increase in stroke volume from a 250 ml fluid bolus. PPV was calculated using a classic method and derived from a generalized additive model (GAM). Results are presented as *estimate* [95% confidence interval].

		GAM PPV				Classic PPV			
Tidal volume [ml kg^{-1}]	Respiratory rate [min^{-1}]	Optimal threshold[1]	Sensitivity	Specificity	AUC	Optimal threshold[1]	Sensitivity	Specificity	AUC
10	10	7.9%	0.80 [0.50;1.00]	0.65 [0.50;0.80]	0.73 [0.57;0.90]	11.4%	0.60 [0.30;0.90]	0.88 [0.78;0.97]	0.74 [0.57;0.92]
8	10	6.9%	0.70 [0.40;1.00]	0.72 [0.56;0.85]	0.68 [0.47;0.89]	8.1%	0.60 [0.30;0.90]	0.82 [0.69;0.92]	0.73 [0.55;0.91]
6	10	5.9%	0.70 [0.40;1.00]	0.74 [0.61;0.87]	0.77 [0.61;0.93]	5.8%	0.90 [0.70;1.00]	0.74 [0.61;0.87]	0.82 [0.66;0.98]
4	10	3.1%	0.80 [0.50;1.00]	0.50 [0.35;0.65]	0.67 [0.47;0.86]	4.9%	0.80 [0.50;1.00]	0.78 [0.62;0.90]	0.79 [0.62;0.96]
8	17	5.7%	0.90 [0.70;1.00]	0.62 [0.47;0.78]	0.73 [0.55;0.91]	4.9%	0.90 [0.70;1.00]	0.57 [0.42;0.72]	0.71 [0.54;0.88]
6	17	5.7%	0.70 [0.40;1.00]	0.82 [0.68;0.92]	0.74 [0.56;0.91]	4.4%	0.90 [0.70;1.00]	0.71 [0.55;0.84]	0.79 [0.62;0.96]
8	24	7.2%	0.80 [0.50;1.00]	0.80 [0.68;0.93]	0.74 [0.51;0.97]	6.7%	0.70 [0.40;1.00]	0.88 [0.78;0.97]	0.79 [0.60;0.98]
6	24	4.6%	0.70 [0.40;1.00]	0.70 [0.55;0.82]	0.70 [0.51;0.90]	4.1%	0.80 [0.50;1.00]	0.72 [0.57;0.88]	0.75 [0.59;0.91]
8	31	7.7%	0.50 [0.12;0.88]	0.94 [0.86;1.00]	0.70 [0.46;0.94]	5.0%	0.75 [0.50;1.00]	0.83 [0.69;0.94]	0.79 [0.56;1.00]
6	31	4.3%	0.70 [0.40;1.00]	0.62 [0.47;0.78]	0.65 [0.45;0.85]	3.1%	0.70 [0.40;0.90]	0.68 [0.53;0.80]	0.62 [0.40;0.84]

[1] Threshold with maximum Youden index.
